# Graphdiyne-modified TiO_2_ nanofibers with osteoinductive and enhanced photocatalytic antibacterial activities to prevent implant infection

**DOI:** 10.1038/s41467-020-18267-1

**Published:** 2020-09-08

**Authors:** Rui Wang, Miusi Shi, Feiyan Xu, Yun Qiu, Peng Zhang, Kailun Shen, Qin Zhao, Jiaguo Yu, Yufeng Zhang

**Affiliations:** 1 State Key Laboratory Breeding Base of Basic Science of Stomatology (Hubei-MOST) and Key Laboratory of Oral Biomedicine, Ministry of Education, School and Hospital of Stomatology, Wuhan University, 430079 Wuhan, PR China; 2 Medical Research Institute, School of Medicine, Wuhan University, 430071 Wuhan, PR China; 3 Foshan Xianhu Laboratory of the Advanced Energy Science and Technology Guangdong Laboratory, Xianhu Hydrogen Valley, 528200 Foshan, PR China; 4 State Key Laboratory of Advanced Technology for Material Synthesis and Processing, Wuhan University of Technology, Luoshi Road 122#, 430070 Wuhan, PR China

**Keywords:** Antimicrobials, Photocatalysis, Biomedical engineering

## Abstract

Titanium implants have been widely used in bone tissue engineering for decades. However, orthopedic implant-associated infections increase the risk of implant failure and even lead to amputation in severe cases. Although TiO_2_ has photocatalytic activity to produce reactive oxygen species (ROS), the recombination of generated electrons and holes limits its antibacterial ability. Here, we describe a graphdiyne (GDY) composite TiO_2_ nanofiber that combats implant infections through enhanced photocatalysis and prolonged antibacterial ability. In addition, GDY-modified TiO_2_ nanofibers exert superior biocompatibility and osteoinductive abilities for cell adhesion and differentiation, thus contributing to the bone tissue regeneration process in drug-resistant bacteria-induced implant infection.

## Abstract (web summary)

Infection is a growing issue in artificial implants and has become an area of significant interest. Here, the authors report on graphdiyne titanium dioxide composite nanofibres with enhanced photocatalytic reactive oxygen species generation and osteoinductive properties, demonstrated in infected implant models.

## Introduction

Since the early 1970s, titanium (Ti) and Ti-alloy implants have been widely applied in orthopedics and dentistry due to their benign biocompatibility, chemical stability, and mechanical properties^1,2^. However, orthopedic implant-associated infections mainly caused by Staphylococcus aureus (S. aureus) delay the healing process and lead to bone loss, requiring extensive surgical intervention, and long-term antibiotic therapy^3^. Repeated antibiotic treatment increases the possibility of drug resistance (40% of pathogenic S. aureus are methicillin-resistant forms)^4^. These infections often lead to implant failure, which requires implant replacements and causes chronic and/or relapsing disease^3,5^. In addition to causing pain and financial loss in further treatment, severe infections might induce amputation or life-threatening sepsis^6^.

Current studies to optimize bone implants mainly focus on surface modification or changing topological features to promote stem cell differentiation and induce bone regeneration^7–11^. However, the antibacterial ability of the implants should also be taken into consideration. Theoretically, TiO_2_ as a photocatalytic material can produce reactive oxygen species (ROS) to kill bacteria under UV irradiation^12^, while the recombination of generated electrons and holes reduces the photocatalytic performance of TiO_2_ and restricts its practical antibacterial effects^13^. Recently, researchers have loaded antibiotics, metal nanoparticles, and other antimicrobial elements onto TiO_2_ surfaces for antibacterial applications^13–16^. However, the bacterial resistance arising from antibiotic application and the potential cytotoxicity of metal nanoparticles limit their clinical applications. Therefore, an ideal titanium implant should have features of a controllable antibacterial surface based on steady long-term tissue regeneration guidance properties.

As a new member of 2D carbon-based nanomaterials, graphdiyne (GDY) has been predicted to be the most stable diacetylenic carbon allotrope^17^. It is composed of 18-C hexagon units with benzene rings connected by butadiyne linkages (–C≡C–C≡C–). Compared with graphene, the particular *sp* and *sp*^2^ hybridized carbon atoms of GDY endow the latter with a unique electronic structure and superior electrical conductivity, which are highly beneficial for electron transfer and enhance the catalytic effects of metals and their oxides^18–23^. In addition, GDY and its derivatives exhibit great stability and biocompatibility in some in vivo studies^24–26^, thus displaying favorable biological properties in biosensing and cancer therapy. Oxidative GDY alone was also reported to have antibacterial effect under visible light through ROS generation, direct contact and good dispersion^27^. In addition, graphene, the most investigated allotrope of GDY, possesses favorable osteogenic capability, indicating that GDY also has osteoinductive potential^28^. In view of the aforementioned advantages, GDY is an ideal material to coordinate the dual functions of TiO_2_ implants with both tissue regeneration properties and antibacterial effects.

In this study, we synthesize a TiO_2_/graphdiyne composite (TiO_2_/GDY) to improve its bioactivity. By changing the surface charges of GDY sheets and TiO_2_ nanofibers, we successfully assemble GDY onto TiO_2_ by electrostatic force. TiO_2_/GDY nanofibers exhibite excellent photocatalytic performance with increased photocatalytic ROS production. The abundant generated ROS induce cellular component oxidation and bacterial cell wall perforation, which lead to membrane leakage, structural destruction, and eventual bacterial death. Furthermore, the antibacterial effect of TiO_2_/GDY is prolonged with surprising sustained ROS release that prevents Methicillin-resistant Staphylococcus aureus (MRSA) biofilm formation. In addition, GDY enhances the biocompatibility of TiO_2_ nanofibers and exhibits the adsorption of osteoinductive components in vitro, thus promoting cell proliferation and osteoinduction abilities. Mouse orthopedic implant infection models further demonstrate the excellent sterilization and bone regeneration effects of TiO_2_/GDY nanofibers. Due to their great multilevel properties, TiO_2_/GDY nanofibers can be applied as an ideal scaffold or surface modification strategy for titanium implants to prevent implant infections and promote bone tissue regeneration.

## Results

### Characterization and photocatalytic properties of TiO_2_/GDY

**Fig. 1:**
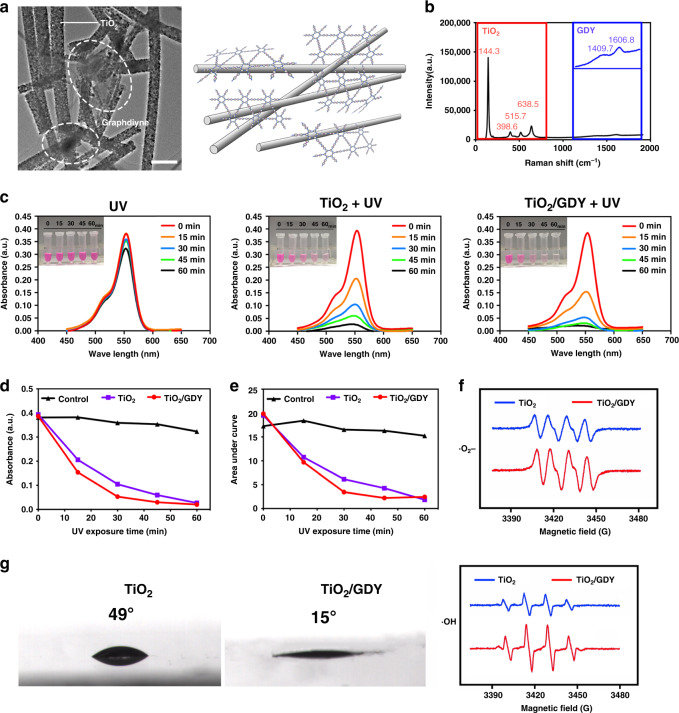
Characterization and photocatalytic properties of TiO_2_/GDY. **a** Transmission electron microscopy image of TiO_2_/GDY (left, scale bar = 0.5 μm) (*n* = 3 separate materials) and illustration of the TiO_2_/GDY structure (right). **b** Raman spectra of TiO_2_/GDY. The main peaks of TiO_2_ are shown in the red box, and the magnified peaks of GDY are shown in blue. **c** Absorption curves and photos of rhodamine B degradation by TiO_2_ and TiO_2_/GDY photocatalysis under UV irradiation for different times (0, 15, 30, 45, and 60 min). **d** Trends in the absorbance peaks of the RhB curves at each UV exposure time point. **e** Areas under the absorption curves at each time point. **f** Electron spin resonance for •OH (below) and •O_2_^−^ (above) generation after TiO_2_ (blue) and TiO_2_/GDY (red) were induced by UV. **g** Contact angle measurements of TiO_2_ and TiO_2_/GDY nanofibers. Source data of **b**–**f** are provided as a Source Data file.

First, we used transmission electron microscopy (TEM) to show the microstructure of the prepared TiO_2_/GDY. The TEM image confirms that the TiO_2_ nanofibers and GDY were successfully self-assembled and that the diameter of the nanofibers was ~150 nm (Fig. 1a). Then, we used Raman spectroscopy to confirm the molecular component of TiO_2_/GDY. As shown in Fig. 1b, four main peaks in red from 144.3 to 638.5 cm^−1^ represent TiO_2_, while the two peaks of 1409.7 and 1606.8 cm^−1^ magnified in blue are ascribed to the aromatic bonds, the C–C triple bonds and their conjugate bond neighbors of GDY^29^.

The photocatalytic activity of TiO_2_/GDY and TiO_2_ nanofibers was measured through a rhodamine B (RhB) degradation assay. RhB belongs to xanthene dyes, a class of stable fluorescent dyes that can be degraded by the photocatalytic effect of TiO_2_ through photobleaching and N-desulfurization^30^. After the TiO_2_/GDY or TiO_2_-mixed RhB solution was irradiated with ultraviolet (UV) light (365 nm, 2 W cm^−2^) for 60 min, the solution became paler in color, and its absorbance decreased (Fig. 1c). According to the variations in absorption peaks and area under the curve, the RhB photodegradation with TiO_2_/GDY was faster than that with TiO_2_ due to the capability of electron separation of GDY (Fig. 1d, e). Conversely, pure RhB irradiated under UV light barely degraded. Furthermore, the electron spin resonance (ESR) spectra show that the signals of ·OH, ·O_2_^−^, 1O_2_, and ·OOH derived from TiO_2_/GDY were stronger than those from TiO_2_ under UV irradiation, demonstrating that the ROS (·OH, ·O_2_^−^, 1O_2_, and ·OOH) generation ability of TiO_2_ nanofibers was significantly enhanced after GDY assemblage (Fig. 1f and Supplementary Fig. 1). Also, photocatalytic TiO_2_/GDY produced more H_2_O_2_ than TiO_2_, and maintained a relative high concentration level in at least 60 min after Xe lamp irradiation (Supplementary Fig. 2). In addition, the contact angle of TiO_2_/GDY was 15°, which was much smaller than that of TiO_2_ (49°) (Fig. 1g).

### Morphology and viability of cells on TiO_2_/GDY

**Fig. 2:**
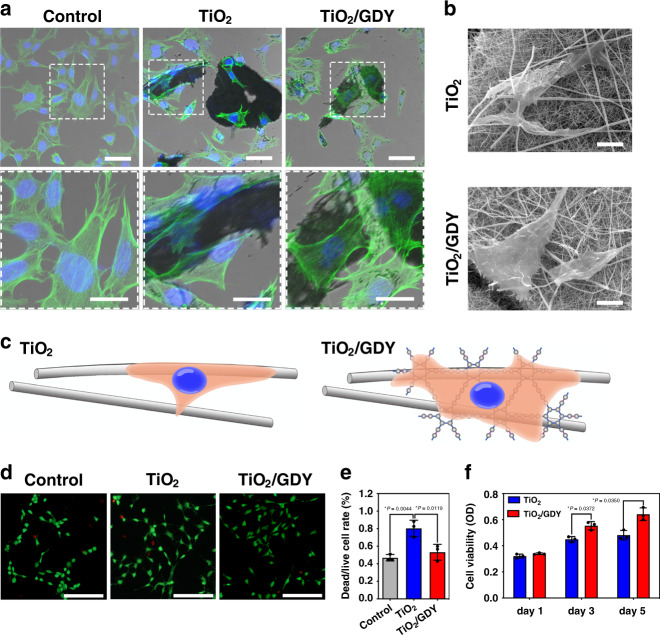
Cell morphology and viability of TiO_2_/GDY and TiO_2_. **a** Cytoskeleton staining of MC3T3-E1 cells with FITC-tagged phalloidin (green) and DAPI (blue) after co-culturing with TiO_2_ or TiO_2_/GDY nanofibers for 24 h; scale bar = 50 μm (above); 25 μm (below) (*n* = 3 independent experiments). **b** SEM images showing the MC3T3-E1 cell morphology on nanofibers (scale bar = 10 μm).(*n* = 3 independent experiments). **c** Illustration of the states of cell adhesion on different nanofibers. **d** Live/dead staining with calcein AM (green) for live cells and PI (red) for dead and apoptotic MC3T3-E1 cells cultured with nanofibers (scale bar = 50 μm). **e** Ratio of PI- and calcein AM-stained positive cells quantitated from the live/dead stain. (*n* = 3). **f** Cell proliferation assay for MC3T3-E1 cells cultured with TiO_2_ or TiO_2_/GDY nanofibers for 1, 3, and 5 days. The data of **e**, **f** are shown as the mean ± S. D., error bars = Standard Deviation (*n* = 3 independent experiments, one-way ANOVA was applied for statistical analysis, *p < 0.05; **p < 0.01). Source data of **e**, **f** are provided as a Source Data file.

To investigate the biocompatibility of nanofibers during bone regeneration, murine-derived osteoblast-like MC3T3-E1 cells were used for in vitro evaluation. The cells were cultured with TiO_2_/GDY or TiO_2_ for 24 h, and then the cytoskeletons were stained with fluorescein isothiocyanate (FITC)-phalloidin to observe the cell morphology. Compared with those cultured with TiO_2_, cells on TiO_2_/GDY nanofibers tended to aggregate and adhere more. The attached cells exhibited more spreading, filopodia extension and intercellular contacts than did the normal cultured cells in the control group (Fig. 2a). The cell area calculation also showed that the cells on TiO_2_/GDY was more spreading than on TiO_2_ (Supplementary Fig. 3a). The SEM images indicate that cells could adhere to both TiO_2_ and TiO_2_/GDY. However, their adhesion and spreading were flatter and tighter on TiO_2_/GDY than on TiO_2_ (Fig. 2b). As shown in the illustration (Fig. 2c), cells tended to spread on TiO_2_/GDY for the expansive contact area, and the increased hydrophilicity of the surface was attributed to GDY. In addition, there were more cells adhered on TiO_2_/GDY than on TiO_2_ (Supplementary Fig. 3b).

The live/dead staining and CCK-8 assays indicated that TiO_2_/GDY barely led to cell death and inhibited cell proliferation (Fig. 2d–f). TiO_2_/GDY produced few dead cells (red) with or without UV irradiation, while TiO_2_ nanofibers induced cell death without UV irradiation and caused a significant rise with UV irradiation (Fig. 2d, e; Supplementary Fig. 4). The CCK-8 assay on TiO_2_/GDY with different GDY weight ratios revealed that TG0.5 used in this research showed good biocompatibility even at high concentrations (Supplementary Fig. 5). In summary, these results demonstrated that TiO_2_/GDY exhibited favorable cell adhesion and biocompatible characteristics that contributed to cell proliferation and migration on the scaffold for bone regeneration.

### Photocatalytic antibacterial effects of TiO_2_/GDY in vitro

**Fig. 3:**
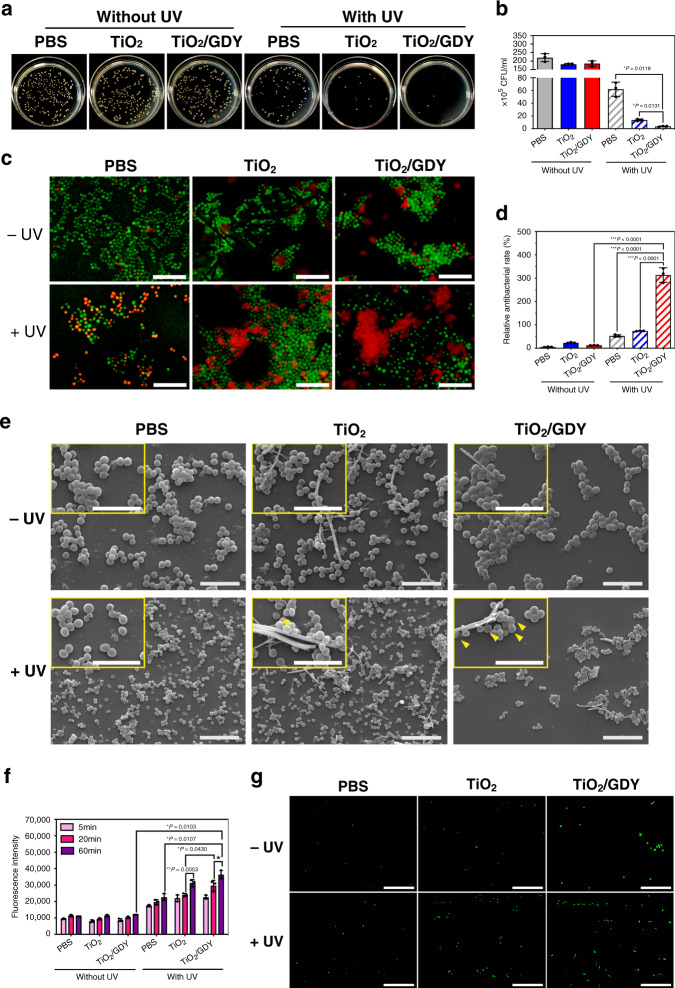
Photocatalytic antibacterial effects of TiO_2_/GDY and TiO_2_ in vitro. MRSA were treated with TiO_2_, TiO_2_/GDY or PBS, with or without UV (365 nm, 2 W cm^−1^) irradiation. **a**, **b** Photos of agar plates and quantitative analysis of bacterial colonies. **c** Live/dead stained images of MRSA biofilms with photocatalytic treatment (scale bar = 10 μm). **d** Semiquantification of the live/dead staining for the ratio of PI- and calcein AM-stained areas. **e** SEM images of MRSA biofilms after exposure to photocatalytic treatment with nanofibers; yellow arrowheads in the magnified inset images indicate holes on the bacterial surface; scale bar = 5 μm (upper), 10 μm (below). **f** Quantitative analysis of radical oxygen species (ROS) in MRSA 5, 20, and 60 min after photocatalytic treatment. **g** Fluorescence images of ROS in bacteria for 24 h (FITC-tagged probes); scale bar = 100 μm. The data of **b**, **d** and **f** are shown as the mean ± S. D., error bars = Standard Deviation (*n* = 3 independent experiments, one-way ANOVA was applied in **b**, **d**, and multiple *t* test was applied in **f** for statistical analysis, *p < 0.05; **p < 0.01; ***p < 0.001). Source data of **b**, **d**, **f** are provided as a Source Data file.

According to recent statistics, nearly 40% of S. aureus-caused orthopedic implant-associated infections are methicillin-resistant forms, and the constant rise of new resistance mechanisms increases the difficulties of infectious disease treatment^4,31^. Thus, we used MRSA to explore the antibacterial ability of TiO_2_/GDY and develop a drug-resistant bacterium-associated implant infection model. Standard plate counting assay results showed that after pretreatment with the nanofibers and irradiation with UV, the number of live bacteria decreased significantly. The number of colonies of the TiO_2_/GDY + UV group reduced by 98% compared to that of the group not treated with UV (Fig. 3a, b). In particular, TG0.5 exhibited the best antibacterial effect among various GDY proportions of nanofibers (Supplementary Fig. 6). Moreover, we compared the oxide graphene (GO) assembled TiO_2_ nanofibers (TiO_2_/GO) with TiO_2_/GDY. It revealed that the colonies in photocatalytic TiO_2_/GO treated group were twice as much as in photocatalytic TiO_2_/GDY group and were much bigger, indicating TiO_2_/GDY nanofibers got better sterilization effect after UV irradiation. Colonies in GO + UV and GDY + UV group were slightly higher than PBS + UV group, as a result, the effects of GO or GDY alone were also excluded (Supplementary Fig. 7).

Because biofilm formation plays a pivotal role in the pathogenesis of implant infections^3^, we then investigated the biofilm eradication effect of TiO_2_/GDY. By performing a live/dead staining assay on MRSA biofilms after photocatalytic treatments, we found that TiO_2_/GDY after UV irradiation destroyed approximately 76% of the biofilm (Fig. 3c). The ratio of dead cells to live cells in the TiO_2_/GDY + UV group was 4 times higher than that in the TiO_2_ + UV group (Fig. 3d). Next, we investigated whether photocatalytic TiO_2_/GDY could also prevent biofilm formation. After pretreatment with photocatalysis, MRSA cells were cultured for 24 h on slides and fixed for SEM scanning and crystal violet staining. SEM images of the TiO_2_/GDY + UV group show a few single colonies, while those of other groups present several clusters with multiple colonies. The semiquantitative evaluation by crystal violet staining also revealed that TiO_2_/GDY + UV group got the least biofilm formation (Fig. 3e, Supplementary Fig. 8). Furthermore, in the magnified SEM images, pits are observed on the photocatalysis-treated group, especially in TiO_2_/GDY with UV irradiation. We attributed those pits to the cell wall perforation caused by ROS^32^.

Then, we detected the intracellular ROS production capacity of TiO_2_/GDY-treated bacteria by a fluorescent ROS probe (DCFH-DA). As shown in Fig. 3f, g, GDY modification increased ROS generation in MRSA under UV irradiation compared with that in other groups. In addition, ROS release after photocatalysis treatment was sustained for at least 60 min in the TiO_2_/GDY + UV group (Fig. 3f). The above results proved that photocatalyst TiO_2_/GDY could not only damage existing biofilms but also prevent biofilm formation. The SEM scanning of single bacteria at 0, 1 h after photocatalytic treatment indicated that the TiO_2_/GDY + UV treatment destroyed the cell structure of MRSA (Supplementary Fig. 9a). Due to the small amount of ROS generation by near-UV irradiation, PBS + UV treatment also induced cell wall perforation but not as severe as TiO_2_/GDY + UV. Moreover, the decrease of intracellular adenosine triphosphate (ATP) generation of TiO_2_/GDY + UV group indicating that the cell metabolism was disrupted (Supplementary Fig. 9b).

### Osteogenic effect of TiO_2_/GDY and TiO_2_ in vitro

**Fig. 4:**
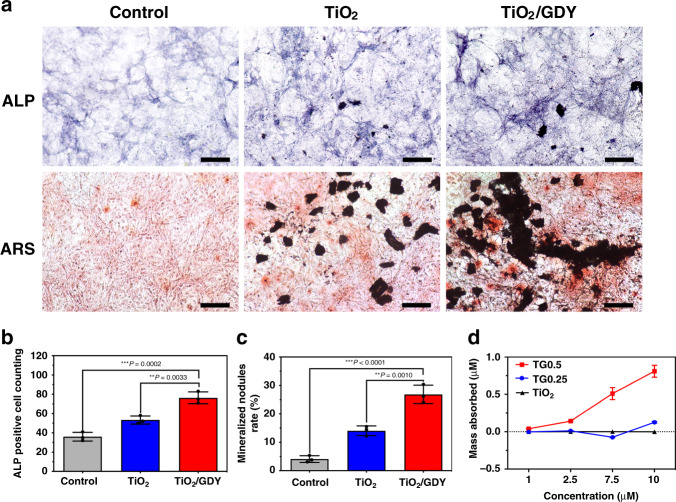
Osteogenic effect of TiO_2_/GDY and TiO_2_ in vitro. MC3T3-E1 cells were cultured with TiO_2_, TiO_2_/GDY or PBS under osteogenic induction. **a** Alkaline phosphatase activity (upper labeled ALP) and Alizarin Red S staining (lower labeled ARS) on day 14. **b**, **c** Semiquantitative analysis of ALP activity and ARS staining. **d** Loading capacity of dexamethasone on TiO_2_/GDY (TG0.25, TG0.5) and TiO_2_ nanofibers for 24 h. Scale bar = 100 μm. The data are shown as the mean ± S.D., error bars = Standard Deviation (*n* = 3 independent experiments). One-way ANOVA was applied for statistical analysis in **b**, **c** (*p < 0.05; **p < 0.01; ***p < 0.001). Source data of **b**–**d** are provided as a Source Data file.

To study the osteoinductive properties of the nanofibers in vitro, MC3T3-E1 cells were treated with TiO_2_/GDY or TiO_2_ nanofibers and cultured for 14 days. Alkaline phosphatase (ALP) is the most widely recognized biochemical marker for osteoblast activity during osteogenic differentiation^33^. The ALP staining assay showed that the TiO_2_/GDY-treated group exhibited a high ALP level (Fig. 4a, b). Alizarin Red S (ARS) staining revealed that the TiO_2_/GDY-treated MC3T3-E1 cells had nearly twofold more calcium deposition and mineralization nodules than did the TiO_2_ group (Fig. 4a, c). As comparison, GDY showed a slightly increase in ALP expression than GO, which contributed to the better osteoinductive ability of GDY-assembled TiO_2_. In addition, the pretreating of UV irradiation on materials has no significant effect on cell osteogenic differentiation (Supplementary Fig. 10).

Studies have shown that graphene has the ability to preconcentrate osteoinducers such as dexamethasone and β-glycerophosphate to promote osteogenic differentiation^34^. Therefore, we asked whether GDY modification acted in the same mechanism. We determined the capacities of TG0.5, TG0.25, and TiO_2_ nanofibers to load dexamethasone by UV spectrophotometry. The adsorption isotherms showed that TG0.5 had the highest amount of dexamethasone adsorption after 1 day of incubation (Fig. 4d). The adsorbed dexamethasone can induce the expression of proteins and enzymes required during bone differentiation, thus leading to increased mineral deposition^35^. These results confirmed that TiO_2_/GDY nanofibers were equipped with outstanding osteoinductive properties and deserve to be an ideal implant material for bone regeneration. ALP, Osteocalcin (OCN), Osterix (OSX), and Collagen 1a (Col1a) are all osteogenetic markers in early stage of bone remodeling^36,37^. Real time-qPCR assay revealed that both TiO_2_/GO and TiO_2_/GDY could enhance the expression of osteogenesis factors which promoted osteoblast differentiation. While the TiO_2_/GDY treatment showed a better enhancement on ALP expression (Supplementary Fig. 11), which was also convinced by the ALP staining.

### Antibacterial and osteogenic effects of TiO_2_/GDY in vivo

**Fig. 5:**
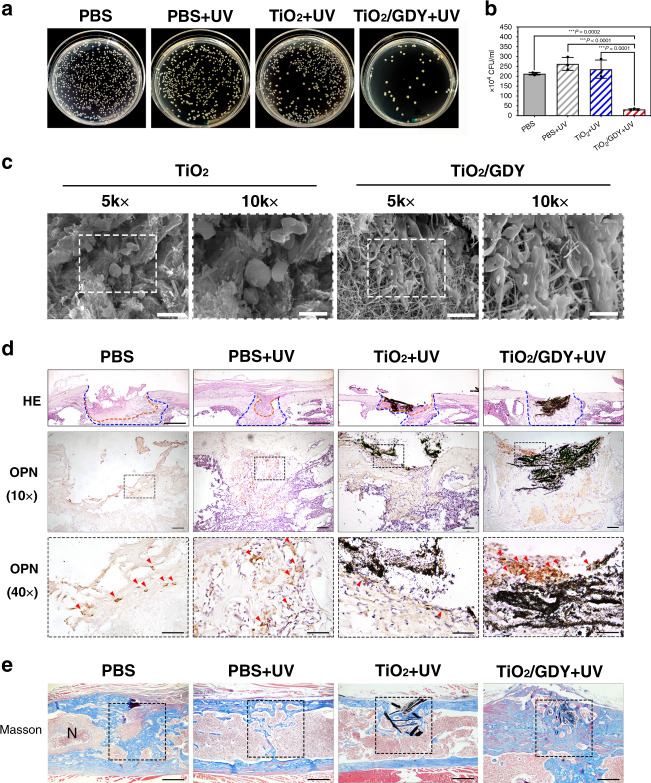
Antibacterial and osteogenic effects of TiO_2_/GDY in vivo. **a**, **b** Photographs and quantitative analysis of the bacterial colonies of the infected femurs treated with TiO_2_, TiO_2_/GDY, or PBS as a control, with UV irradiation (365 nm, 2 W cm^−1^, 5 min). The data are shown as the mean ± S.D., error bars = Standard Deviation (*n* = 3 biological independent samples, one-way ANOVA was applied for statistical analysis, *p < 0.05; **p < 0.01; ***p < 0.001). **c** SEM images of the mouse femur tissues with TiO_2_ or TiO_2_/GDY nanofiber implantation after 5 days, with ×5000 (left) and ×10,000 (right) magnification, scale bar = 10 μm (left), 5 μm (right). (*n* = 3 biological independent samples). **d** H&E staining and immunohistochemical staining of infected tissues after 5 days. Orange dotted lines in the H&E images represent infectious and necrotic areas; between the orange and blue dotted lines are new bone formation areas (scale bar = 500 μm). OPN 40×: scale bar = 50 μm; OPN 10×: scale bar = 100 μm, and red arrowheads point out OPN-positive osteoblasts. (*n* = 3 biological independent samples). **e** Masson staining for bone formation after 4 weeks (scale bar = 500 μm). “N” in the PBS image represents the necrotic tissue in the bone marrow. (*n* = 3 biological independent samples.) Source data of (**b**) are provided as a Source Data file.

After confirming the antibacterial and osteoinductive properties of TiO_2_/GDY in vitro, we then established implant-associated infection models to verify the nanofibers’ in vivo therapeutic effects. Five days after surgery, we counted the bacterial colonies in the grinding fluid of the implanted femur. Plate counting results showed that there was 85% less MRSA in TiO_2_/GDY + UV-treated femurs than in the PBS group, better than GO + UV, GDY + UV, TiO_2_/GO + UV, and TiO_2_ + UV group (Fig. 5a, b; Supplementary Figs. 12, 13), which indicated that TiO_2_/GDY could effectively inhibit bacterial infection with UV irradiation under the condition of MRSA contamination during implantation.

To explore the in vivo cell compatibility of nanofibers, we scanned the implanted nanofibers by SEM 5 days after implantation (Fig. 5c). Consistent with the in vitro observations, cells appeared to attach firmly on the surface of the GDY-assembled nanofibers. Then, we used hematoxylin–eosin (H&E) staining to reveal the pathological features of femur infections. Figure 5d shows that the PBS, PBS + UV and TiO_2_ + UV groups all had significant infected and necrotic zones (orange dotted lines) in the bone defect area, while the TiO_2_/GDY + UV group showed minimal inflammatory cell infiltration. Furthermore, the TiO_2_/GDY + UV group had the widest new bone regeneration area (blue dotted lines, Fig. 5d and Supplementary Figs. 14 and 15). Immunohistochemical assays on the expression of the osteogenesis marker osteopontin (OPN) revealed many OPN-positive cells in the TiO_2_/GDY-implanted femurs (Fig. 5d and Supplementary Fig. 14). The results above all confirmed that TiO_2_/GDY provided a good osteoinductive microenvironment in vivo. In contrast, without UV irradiation, the femurs barely exhibited new bone formation and were filled with infectious tissue (Supplementary Fig. 14a), which proved the vital role of the antibacterial process during bone infections.

Four weeks after surgery, we conducted Masson staining to evaluate the bone regeneration status. The PBS group still had an unhealed hole and chronic infection with necrotic tissue in the bone marrow (Fig. 5e). However, the bone defect implanted with TiO_2_/GDY was completely healed with the generation of large amounts of new bone (blue staining in dashed boxes). In comparison, the bone masses of the PBS + UV and TiO_2_ + UV groups were inadequate, and the process of bone regeneration was very slow (Supplementary Fig. 16). H&E staining images also exhibit the bone remodeling levels of different groups (Supplementary Fig. 17). Both GDY + UV and GO + UV group got great new bone formation surrounded the implants but there were still unhealed bone defects. We attributed this to the insufficient bactericidal effects and non-supporting structure in the absence of TiO_2_ nanofibers. In addition, organs in mice were collected and observed with H&E staining for histological toxicity evaluation (Supplementary Fig. 18). There was no significant difference between nanofiber-implanted groups and the PBS control group. It reveals that the systematic toxicity of nanofibers could be neglected. Taken together, the in vivo studies verified the combined outstanding bactericidal and osteogenic properties of TiO_2_/GDY in implant-associated infections.

## Discussion

Implant device-associated infections are a series of typical complications affecting the prognosis of orthopedic surgery. Titanium implants may become loose and displaced with delayed healing afterwards. Furthermore, bacteria spreading within the entire body lead to local bone loss, amputation, systemic inflammation, and even sepsis^3,5,6^. Although TiO_2_ itself has a certain degree of antibacterial effects when induced by UV light, the effects are limited since the photocatalytic effect of TiO_2_ alone is extremely transient^12,38^. Researchers have introduced antibiotics and metal nanoparticles onto titanium implant scaffolds to enhance bacteriostatic efficacy, but these studies still exhibit various drawbacks^14,15^. In this study, we changed the zeta potential of TiO_2_ nanofibers to positive and the charge of a novel two-dimensional carbon-based nanomaterial, GDY, to negative to synthesize a GDY-combined TiO_2_ heterojunction to enhance the photocatalytic effect^39,40^. Further, our previous XPS results indicated that GDY contained oxyfunctional groups, such as C–OH and C=O^23^. After the composites were calcined at 350 °C for 2 h, a strong interaction was produced between TiO_2_ and GDY due to their chemical action, hydrogen bonding and van der Waals force. This results in the composites with high structure stability in vivo experiments.

The TEM image and chemical bond analysis of the Raman spectra illustrated that the TiO_2_/GDY compound was successfully synthesized with a GDY sheet attached to the surface of TiO_2_ nanofibers. TiO_2_/GDY maintained the nanostructure with a large specific surface area and a porous network. In addition to its typical application as an orthopedic implant material, TiO_2_ is also a widely developed semiconductor metal oxide with photocatalytic performance^41^. With UV irradiation, electrons are excited from the valence band to the conduction band, producing electron–hole pairs. Free electrons migrate to the surface of TiO_2_ and participate in redox reactions, producing ROS such as hydroxyl radicals, superoxide anions, and hydrogen peroxide (H_2_O_2_)^12^. However, the high recombination rate of electrons and holes in TiO_2_ restrains its photocatalytic antibacterial effect. Studies have been conducted to improve the photocatalytic efficiency of TiO_2_, such as by reducing the particle size^42^, modifying the nanoscale surface, and doping with Cd or other elements^43–45^. As the only two-dimensional carbon nanomaterial containing both *sp* and *sp*^2^ hybrid carbon atoms, GDY has a rigid carbon mesh and a natural band gap, which result in a large specific surface area and high hole mobility^22,23,40,46^. The superior conductive GDY assemblage made the free electrons generated on TiO_2_ under UV irradiation easily adsorb on the surface of GDY, thereby reducing the recombination rate and intensifying the photocatalytic performance. As evidence, RhB degraded more rapidly by photocatalytic TiO_2_/GDY than by pure TiO_2_. In addition, the generation of ROS including ·OH, ·O_2_^−^, 1O_2_, ·OOH, and H_2_O_2_ was also detected through ESR and ferric ion titration method. Oxidative cellular damage or oxidative stress occurs when the oxidation ability of ROS overpowers that of antioxidants in the living organisms. If the balance between the generation and the detoxification of ROS is disturbed due to oxidative stress, the viability of the cells would decrease. ROS inhibit bacteria through interference of bacteria metabolism, cell wall proliferation and induction of DNA damage^47,48^. For example, hydroxyl radicals can attach to cell walls and consequently cause cell destruction through the rapid release of potassium ions or the oxidization and degradation of intracellular CoA enzyme^12,49^. On SEM images in this research, pits were found on the bacterial surface with photocatalytic treatment (Fig. 3e). After 1 h UV irradiation, bacterial structure was destructed (Supplementary Fig. 9a). In addition, as ATP is the essential energy source of cell metabolism generated from respiratory action, the lowest ATP level in TiO_2_/GDY + UV group exhibited that the bacterial metabolism was significantly inhibited (Supplementary Fig. 9b).

A primary problem of medical device-associated infections, biofilms are responsible for the persistence of implant infections and are a source of bacterial dissemination to other sites in the body^3,50^. In this study, the biofilm staining assay of MRSA demonstrated that TiO_2_/GDY nanofibers could not only effectively eliminate bacteria in the mature biofilms but also inhibit biofilm formation (Fig. 3c, e). In the implant-associated infection models, no local necrotic infection area was observed in femurs implanted with UV-irradiated TiO_2_/GDY where the bone tissue was well repaired (Fig. 5d). Although TiO_2_/GDY did not exhibit remarkable improvement in the photocatalytic antibacterial effect compared to that exhibited by TiO_2_ nanofibers in vitro, the combined characteristics of TiO_2_/GDY made it much more efficient for in vivo treatment.

In the antibacterial experiment, a low dose of near-ultraviolet light (365 nm, 2 W cm^−1^) was applied within a short time. Because near-ultraviolet light has a sterilization effect by damaging DNA and generating ROS^51^, the group with UV irradiation alone showed a certain antibacterial effect in vitro. However, the bactericidal effect of low-dose UV light alone was far lower than the synergistic effect with nanofibers. In addition, the cell viability assay showed no cytotoxicity of TiO_2_/GDY on eukaryotes under irradiation, indicating favorable biocompatibility of the TiO_2_/GDY treatment. The sterilization effect of oxygen radical (such as: ·OH, ·O_2_^−^, 1O_2_, ·OOH) is transient in general. However, the produced H_2_O_2_ has long lifetime and continuous bactericidal effect after the UV was turned off^52^. Therefore, it is not surprising that a sustained antibacterial effect was observed after employment. For in vivo application, we treated MRSA-contaminated nanofibers with UV irradiation before implantation to completely eliminate pathogens and avoid unnecessary tissue damage caused by UV irradiation.

In addition to antibacterial properties, ideal tissue engineering scaffolds with great osteoinductive properties are also required. GDY-based quantum dots were reported to be a potential new fluorescence reagent for imaging and theranostics, which exhibited high biocompatibility and bioactivity both in vitro and in vivo^53^. It has been reported that the high surface energy of a hydrophilic surface can strengthen the interaction with cells and proteins in the early stage of tissue regeneration^54^. Carbon-based nanomaterials have been composited as an effective strategy to improve membrane surface hydrophilicity^55–57^. Accordingly, GDY-assembled TiO_2_ nanofibers showed improved hydrophilicity (Fig. 1g). This is due to the hydrophilic functional groups linked to the *sp* and *sp*^2^ hybridized carbon atoms of GDY^24^. In addition, the 2D and porous structure of GDY contributes to water diffusion^23^. The scaffold material surface with high hydrophilicity favors cell adhesion and proliferation. The cytoskeleton staining, SEM images and cell proliferation assays showed that TiO_2_/GDY nanofibers were beneficial to cell adhesion and proliferation (Fig. 2). The SEM results of the implant materials in vivo further verified the excellent guidance property of TiO_2_/GDY for cell adhesion (Fig. 5c). Although MRSA might tend to adhere to the nanofibers, ROS produced on the materials through photocatalytic effects can effectively eliminate the bacteria in an instant.

In addition, GDY-assembled TiO_2_ nanofibers exhibited improved biocompatibility. The SEM images and live/dead staining of osteoblasts reveal that TiO_2_ nanofibers alone were cytotoxic to some extent, leading to abnormal cell morphology and membrane perforation (Fig. 2a, b). With a kind of high aspect ratio nanostructure, TiO_2_ nanofibers were reported to initiate incomplete cell phagocytosis, raise the expression of inflammatory cytokines, and promote the oxidative stress of cells to cause apoptosis^58,59^. GDY as a kind of two-dimensional polymers, is flexible and scan cover the surface of TiO_2_ nanofibers. As a result, it could improve the interaction between TiO_2_ nanofibers and biological tissues. In addition, due to the unique conjugate π bond system and conjugate diacetylene bond, GDY has been shown to have a long-term scavenging effect on intracellular free radicals^60^. However, our results revealed that photocatalytic TiO_2_/GDY still produced significant ROS generation and sterilization effects. We inferred that the free radical scavenging effect of the GDY component in the nanofibers was nearly negligible compared with the robust photocatalytic effect under irradiation in a very short time. In contrast, during the long-term implantation period in bone tissues, the free radical scavenging property made the nanofibers less cytotoxic than pure TiO_2._ As a result, TiO_2_/GDY not only has photocatalytic antibacterial effects on ROS production but also possesses long-term tissue regeneration guidance properties.

Studies have revealed that the expression of osteogenic genes increases on more hydrophilic surfaces^61^. In addition, some carbon-based nanomaterials, such as graphene, could promote osteogenic differentiation through protein or osteoinductive component adsorption^62^. In this research, GDY assemblage improved the surface hydrophilicity of nanofibers and increased the absorption of small molecules due to the conjugated π bond system. With the increased ratio of GDY in the TiO_2_/GDY compound, the adsorption of dexamethasone, a main effective molecule for osteogenic differentiation, became stronger. In the implant-associated infection model, the TiO_2_/GDY-implanted group showed a significant increase in OPN expression on the fifth day and exhibited the richest new bone formation on the fourth week after implantation in vivo. Due to the negative potential of GDY surface, it would be beneficial to calcium deposition^63^. In addition, GDY nanosheets possess a high surface area, suggesting that they have a better drug loading capacity^64^. These indicated that the specific structure of GDY significantly improved the osteoinductive ability of TiO_2_ nanofibers. In the early stage of osteogenesis, progenitors differentiating into mature-osteoblasts requires varies of transcription factors, including Runx2 and Osterix. Then the differentiated osteoblasts will activate Col1 and OCN genes to secrete matrix such as collagen fibrils. During late stage of mineralization, OCN has a high affinity toward bone and extracellular matrix to induce the calcium deposition. Meanwhile, ALP is able to hydrolyze pyrophosphates (PPi) to generate inorganic orthophosphates (Pi), which participate well in phosphorus metabolism and late biomineralization^36,37^. The enhanced expressions of Col1a, OCN, OSX, and ALP indicate that TiO_2_/GDY could improve osteoblast differentiation and bone mineralization (Supplementary Fig. 11).

**Fig. 6:**
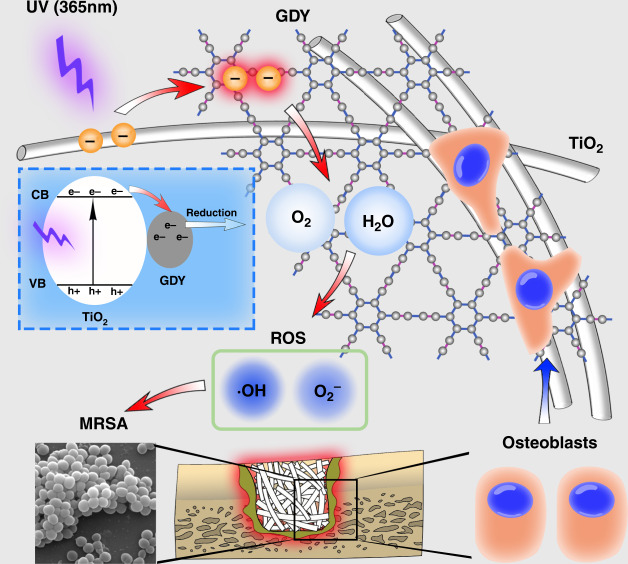
Schematic of the dual function of TiO_2_/GDY in orthopedic implant infection. On the one hand, electrons transferred from UV (365 nm)-irradiated TiO_2_ nanofibers to GDY resulted in more reactive oxygen species generation for sterilization. On the other hand, osteoblasts tended to adhere to the nanofibers due to the great biocompatibility of GDY. The blue dashed box exhibits the free electron generation and transfer in TiO_2_/GDY. “e−” in the blue dashed box represents negative electron, while “h+” represents positive hole.

In conclusion, we prepared GDY-assembled TiO_2_ nanofibers to intensify their photocatalytic antibacterial effect and long-term osteoinductive ability (Fig. 6). With UV irradiation, the free electrons from the photocatalytic-activated TiO_2_ transferred to GDY surface and had longer lifetime, so that the activated state of TiO_2_ was prolonged with more ROS (including oxygen radical and hydrogen peroxide) generation for sterilization (red arrows). On the other hand, GDY-assembled TiO_2_ nanofibers showed great biocompatibility to promote cell adhesion and bone tissue regeneration (blue arrows). Our study not only provided a difunctional TiO_2_/GDY nanofiber as an ideal bone implant but also suggested an effective modification strategy for titanium implants to prevent implant device-associated infections.

## Methods

### Materials preparation

First, 4 g of tetrabutyl titanate (TBT) and 1.5 g of Polyvinyl pyrrolidine (PVP, *M*_W_ = 1.3 × 10^6^) were added to a mixed solution of ethanol (20 g) and acetic acid (4 g) under magnetic stirring for 5 h. Then, The obtained transparent pale-yellow solution was transferred into a 20 mL syringe. The voltage of electrospinning setup and the feeding rate of solution was 20 kV and 2.5 mL h^−1^, respectively. The distance of needle-to-static collector was 10 cm. The collected TiO_2_ fiber precursor was heat-treated at 550 °C for 2 h in air to fabricate TiO_2_ nanofibers.

GDY used in this work was provided by Institute of Chemistry, Chinese Academy of Science^65^. A zeta potential analyzer was used to measure the zeta potential of TiO_2_ and GDY in water at pH 4, which are +15.1 mV and −19.2 mV, respectively. Typically, TiO_2_ nanofibers (200 mg) were dispersed in water at pH = 4, then, magnetically stirred for 1 h at room temperature. Meanwhile, 10 mg of GDY was dispersed in a mixture solution of water (30 mL) and ethanol (60 mL) under sonication irradiation for 2 h. Afterwards, a certain amount of GDY solution was added into the TiO_2_ suspension by stirring vigorously for 1 h. TiO_2_ and GDY were electrostatically self-assembled. Subsequently, the solvents were removed. the obtained composites were calcined at 350 °C for 2 h. The *x* in “TG*x*” represents the added GDY weight ratio. The TiO_2_/GDY applied in the following experiments was the typical TG 0.5.

### Material characterization and photocatalytic properties

The microstructure of TiO_2_/GDY was characterized by TEM (Titan G2 60-300). The molecular structure was detected by Raman spectroscopy (Renishaw Co. RM1000). The surface contact angles of TiO_2_/GDY and TiO_2_ nanofibers were detected by a contact angle meter (Powereach^®^ JC2000C1).

A RhB degradation assay was carried out to detect the photocatalytic activity of TiO_2_/GDY and TiO_2_ nanofibers. 1 mL of 8 μg mL^−1^ RhB solution and 100 μg of photocatalysts (TiO_2_/GDY or TiO_2_) were added into ep tubes and exposed to 365 nm UV light (2 W cm^−2^ for up to 60 min) (Yinzhu, UVEC-4II, China). Each sample was centrifuged for 5 min at 1000 × *g*. The absorbance of the supernatants was detected by an UV spectrophotometer (SOPTOP, UV2800, China).

The ability of TiO_2_/GDY and TiO_2_ nanofibers to generate ·OH and ·O_2_^−^ was respectively measured in water and methanol under 300 W Xe lamp irradiation using ESR (Bruke, emx nano) at room temperature and 5,5-Dimethyl-1-Pyrroline N-oxide (DMPO) as spin trapping reagent. For measuring the generation of 1O_2_ and ·OOH, 2,2,6,6-tetramethyl-piperidine (TEMP) and DMPO were used as trapping reagent. The measurement was performed in water using ESR (Bruke, a300) at room temperature. The generation of H_2_O_2_ was characterized by ferric ion titration method. Fifteen milligrams of photocatalysts were suspended in 15 mL of H_2_O and ultrasonicated for 5 min. The suspension was firstly bubbled with high-purity O_2_ for 30 min and irradiated with 300 W Xe lamp for 3 h. The H_2_O_2_ concentration was measured every 1 h. After turning off the light, H_2_O_2_ level was also tested continuously in 30 and 60 min.

### Cell morphology and viability

The murine-derived osteoblast-like cells MC3T3-E1 were cultured in α-minimal essential medium (Hyclone, Thermo Fisher Scientific Inc.) supplemented with 10% FBS (FBS, Gibco) and maintained at 37 °C in 5% CO_2_ and 95% air in a humidified incubator.

The MC3T3-E1 cells were incubated on confocal dishes at a density of 6 × 10^4^ cell per dish with 20 μg mL^−1^ TiO_2_/GDY or TiO_2_ nanofibers for 24 h. After washed with phosphate buffered saline (PBS) solution, cells were fixed with 4% paraformaldehyde (PFA) for 10 min at room temperature. Removed PFA and washed with PBS, then cells were permeabilized with −20 °C acetone for 5 min. The F-actin and nuclei were stained with FITC Phalloidin (Yeasen, 40735ES75) and DAPI, respectively and observed by laser scanning confocal microscope (LSCM, Leica-LCS-SP8-STED). 25 of random adhered cell areas of each group were calculated through Image J. Adhered cell number was analyzed by DAPI staining of cell nucleuses.

For SEM scanning, MC3T3-E1 cells were seeded on 24-well cell culture plates at a density of 2 × 10^4^ cells per well with 20 μg mL^−1^ TiO_2_/GDY or TiO_2_ nanofibers and incubated for 24 h. After washing with PBS, cells were fixed with 2.5% glutaraldehyde overnight at 4 °C. The samples were washed 3 times with water, then freeze-dried. Followed by sputter coated with gold, samples were observed by SEM (TESCAN VEGA 3 LMU) with an accelerating voltage of 20 kV and a working distance of 11.7 mm.

The live/dead staining assay was carried out with Calcein-AM/PI Double Stain Kit (Yeasen, 40747ES80). In brief, cells were seeded onto glass coverslips in 24-well cell culture plates at a density of 2 × 10^4^ cells per well. 20 μg mL^−1^ TiO_2_/GDY or TiO_2_ nanofibers were added to cells without UV irradiation, received UV (365 nm, 2 W cm^−2^) irradiation together with cells, or received UV irradiation before addition. After 24 h incubation, the glass coverslips were rinsed with 1 × Assay Buffer for 3 times. Afterwards, the coverslips were incubated with 2 μM Calcein-AM and 1 μM propidium iodide (PI) in 1 × Assay Buffer for 15 min at 37 °C. Images were taken using a fluorescence microscope (Zeiss Axio Imager A2). The numbers of live and dead cells were counted by Image J.

Cell proliferation was evaluated by the cell counting kit-8 (CCK-8) assay. MC3T3-E1 cells were seeded in 96-well cell culture plate with 5 × 10^3^ cells per well and incubated overnight for attachment. Then cells were exposed to various concentrations of TiO_2_/GDY or TiO_2_ nanofibers from 1 to 50 μg mL^−1^. At the time points of 1, 3, 5-day incubation, cells were incubated with the CCK-8 agent (Dojindo, Japan) diluted 1:10 with culture medium for 2 h. Then the absorbances were measured by a microplate reader scanning at 450 nm (PowerWave XS2, BioTek, Winooski, VT, USA). The assays were implemented triplicated with three independent experiments.

### Antibacterial properties in vitro

MRSA USA 300 were cultured in an aerophilic environment in LB broth at 37 °C. The bacterial suspension was diluted to 10^9^ colony forming units (CFU mL^−1^) for experimental use after measuring the absorbance at 600 nm using the UV spectrophotometer.

For bacterial standard plate counting assay, MRSA cells were prepared in 20 mL liquid LB medium into 10^9^ CFU mL^−1^ and then added with various ratios of TiO_2_/GDY, TiO_2_, TiO_2_/GO (100 μg mL^−1^), GO, GDY (50 μg mL^−1^) or PBS as control. After 1 h incubation, cells were irradiated with UV (365 nm, 2 W cm^−2^) for 5 min, then 25 μL bacterial suspensions were spread onto LB agar plates. The images were taken, and the colonies were counted using Image J software after 24 h incubation.

The property of biofilm destruction by photocatalysis was determined through live/dead staining. Five hundred microliters 10^9^ CFU mL^−1^ MRSA suspensions were seeded onto 10% Poly-L-Lysine pretreated glass coverslips and incubated 24 h to obtain biofilms in advance. TiO_2_/GDY or TiO_2_ nanofibers were added onto the biofilms with UV irradiation for 5 min. Then the live/dead staining was carried out with Calcein-AM/PI Double Stain Kit. The glass coverslips were rinsed with 1 × Assay Buffer for 3 times. Afterwards, the coverslips were incubated with 2 μM Calcein-AM and 1 μM propidium iodide (PI) in 1 × Assay Buffer for 15 min at 37 °C. Images were taken using a fluorescence microscope (Zeiss Axio Imager A2). The numbers of live and dead cells were counted by Image J.

The effect on the bacterial biofilm formation was investigated through SEM scanning. 10^9^ CFU mL^−1^ MRSA cells were mixed with 100 μg mL^−1^ TiO_2_/GDY or TiO_2_ nanofibers and exposed to UV for 5 min. Five hundred microliters bacterial suspensions were seeded onto a 10% Poly-L-Lysine pretreated glass coverslip and incubated 24 h to form bacterial biofilm. Then the biofilms were washed with PBS and fixed with 2.5% glutaraldehyde overnight at 4 °C. The samples were washed 3 times with water, then freeze-dried. Followed by sputter coated with gold, samples were observed by SEM (TESCAN VEGA 3 LMU) with an accelerating voltage of 20 kV and a working distance of 10.5 mm.

Crystal violet staining was carried out for semi-amount evaluation of biofilm formation. 10^9^ CFU mL^−1^ MRSA cells were mixed with 100 μg mL^−1^ TiO_2_/GDY or TiO_2_ nanofibers and exposed to UV (2 mW cm^−2^) for 5 min. Ten microliters bacterial suspensions were added to 100 μL LB medium in 96-well plate. After 24 h incubation in 37 °C, the biofilms were rinsed and fixed with 4% of PFA for 10 min. Then crystal violet (Servicebio, China) was added 50 μL per well. After 20 min incubation in room temperature, removed the dye liquor and rinsed with ddH_2_O. Ethyl alcohol was added 100 μL per well for dissolution. Absorbance at 590 nm of each well was measured after 20 min shaking (Molecular Devicesmd, spectramax i3x).

ROS generation in MRSA was tested using a ROS assay kit (Beyotime, China). MRSA cells (10^9^ CFU mL^−1^) were loaded with the fluorescence probe (DCFH-DA) in 37 °C for 20 min. Then the bacteria were incubated with 100 μg mL^−1^ TiO_2_/GDY or TiO_2_ nanofibers and exposed to UV for 5 min. The fluorescence intensity of DCF, denoted the intracellular ROS generation, was measured 5, 20, 60 min after UV irradiation (Molecular Devicesmd, spectramax i3x). Images of fluorescence were observed using the fluorescence microscope (Zeiss Axio Imager A2).

10^9^ CFU mL^−1^ MRSA cells were incubated with 100 μg mL^−1^ TiO_2_/GDY or TiO_2_ nanofibers and exposed to UV for 5 min. ATP levels evaluation was carried out in accordance with the manufacturer’s protocol (Promega, G8320). For bacterial structure observation, 10^9^ CFU mL^−1^ MRSA cells were incubated with 100 μg mL^−1^ TiO_2_/GDY or TiO_2_ nanofibers and exposed to UV for 1 h. Cells were fixed and observed with SEM 0, 1 h after treatment.

### Osteogenesis properties in vitro

MC3T3-E1 cells were cultured in 24-well plates at a density of 5 × 10^4^ cells per well. After 24 h incubation, the culture medium was replaced by the osteogenic differentiation media consisting of 10 mM of β-glycerophosphate, 10 nM of dexamethasone and 0.2 mM of L-ascorbic acid 2-phosphate (Sigma-Aldrich). Then 20 μg mL^−1^ of TiO_2_/GDY, TiO_2_/GO or TiO_2_ nanofibers, 10 μg mL^−1^ of GDY or GO were added into the culture medium. Medium was changed every 2–3 days. After 14 days of culture, ALP staining was performed by an ALP staining kit (Beyotime, China) and the positive cells were counted by the Image J software.

ARS staining was carried out on day 14 after osteoinduction. Using 95% alcohol to fix cells for 10 min at room temperature. Cells were stained with 2% ARS at pH of 4.1 for 30 min in 37 °C. Then washed the cells with ddH_2_O for 3 times. The stained mineralized nodules were observed under a light microscope. Semiquantitative analysis of ARS staining was implemented with Image J software by measuring the red staining areas.

Real time-qPCR assay on ALP, OCN, OSX, Col1a mRNA expression was conducted to explore osteogenesis property. MC3T3-E1 cells were cultured on 10 μg mL^−1^ of GO, GDY, TiO_2_/GO, TiO_2_/GDY or PBS, then cultured in osteogenic differentiation media for 7 days. Total RNA from cultured cells was extracted. After reverse transcription, Real time-qPCR analysis was performed for detection of gene transcriptional levels. The amount of mRNA was normalized to GAPDH. The primer sequences were shown in Table 1.

**Table.1:** Real time-qPCR primer sequences.

Genes	Primer sequences (F/R)
Alp	GCTGATCATTCCCACGTTTT/CTGGGCCTGGTAGTTGTTGT
Ocn	AAGCAGGAGGGCAATAAGGT/TTTGTAGGCGGTCTTCAAGC
Osx	CAACCTGCTAGAGATCTGAG/TGCAATAGGAGAGAGCGA
Col1a	TGGCAAAGACGGACTCAAC/GGCAGGAAGCTGAAGTCATAA
Gapdh	GTGAAGGTCGGTGTGAACGG/TCCTGGAAGATGGTGATGGG

### Loading capacity

To determine the loading capacity of the nanofibers, dexamethasone (Aladdin, China) with concentrations ranging from 1 to 10 μM were prepared in phosphate buffer. Each concentration of the solution (0.5 mL) was mixed with TiO_2_/GDY 0.5, TiO_2_/GDY 0.25 and TiO_2_ nanofibers (1 mg mL^−1^, 0.5 mL) separately, and vortexed in a shaker for equilibration at room temperature. After 24 h, the mixtures were centrifuged (12,000 × *g*, 5 min) to collect the supernatant for spectrophotometric measurement. The adsorption isotherm of dexamethasone was obtained using UV–vis spectroscopy (SOPTOP, UV2800, China).

### In vivo implant infection model

All animal surgical procedures were approved by the Ethics Committee for Animal Research of Wuhan University in China. The mouse implant infection model was operated on Kunming mice (8-week-old, female). MRSA prepared for infection were in mid-exponential grown phase of 10^7^ CFU mL^−1^. After anesthesia with isoflurane, the hindlimb of mice were shaved and disinfected with povidone iodine and alcohol. A small incision was made on the lateral aspect along the femur. The femur was exposed with blunt dissection. A cortical bone defect in 1-mm diameter was made close to the lower end of femur by trephination with MANI needle. Meanwhile, MRSA (1 × 10^7^ CFU mL^−1^) infected 100 μg of TiO_2_/GDY, TiO_2_, TiO_2_/GO nanofibers or 50 μg of GDY or GO were implanted into the defect on femur and then received UV (365 nm, 2 W cm^−2^) irradiation for 90 s or not. Afterwards, carefully sutured and closed muscle fasciae and skin. Five days and 4 weeks after surgery, the femurs were harvested after euthanasia by sodium pentobarbital. The samples were ground in sterile PBS (5 mL) for standard plate counting or fixed in 4% PFA for 24 h at room temperature for histomorphological study. Three animal samples were operated for each experiment per group.

### Histomorphological analysis

H&E staining and Masson staining were carried out for histomorphological analysis. Fixed femur samples were decalcified in 10% EDTA for 4 weeks and changed twice a week. Decalcified samples were dehydrated with alcohol gradient and embedded in paraffin. Serial sections in 4 μm thickness were mounted on slides. Samples were deparaffinized and rehydrated before subjected to H&E staining (MXB, China) and Masson staining (MXB, China) in accordance with the manufacturer’s protocol. The quantitative analysis of Masson staining was performed by the Image J software on 5 regions of interest.

For immunohistochemical staining, slices were incubated with the primary antibody of OPN (Santa cruz, sc-21742, 1:100) overnight at 4 °C and then stained with immunohistochemical kit (MXB, China) according to manufacturer’s protocol. The images were visualized by the light microscope. The quantitative analysis was performed by counting positive cells in 5 of the observed areas with Image J software.

### Statistical analysis

All data were presented as means with standard deviations (SD). The significance analysis between two groups was tested with ANOVA and *t* test with GraphPad Prism software (6.0).

### Reporting summary

Further information on research design is available in the Nature Research Reporting Summary linked to this article.

## Supplementary information

Supplementary Information

Reporting Summary

## Data Availability

Source data that support the findings of this study are avaliable in Figshare with the identifier 10.6084/m9.figshare.12715457. Source data underlying Figs. 1b–f, 2e, f,  3b, d, f,  4b–d,  5b and Supplementary Figs. 1, 2, 3, 4b, 5, 6b, 7b, 8, 9b, 11, 12b, 13b, 14b, 16 are provided with this paper. All data pertaining to this work is available from the corresponding author upon reasonable request. Source data are provided with this paper.

## Source data

Source Data
