# Supplementary material for: Graphdiyne-modified TiO2 nanofibers with osteoinductive and enhanced photocatalytic antibacterial activities to prevent implant infection
Source: Nat Commun. 2020 Sep 8;11:4465. doi: 10.1038/s41467-020-18267-1 (PMC7479592; doi:10.1038/s41467-020-18267-1)
Supplement: Supplementary file 1 — Supplementary Information [file 41467_2020_18267_MOESM1_ESM.pdf]

Supplementary Information

Graphdiyne-Modified TiO_2 Nanofibers with Enhanced Photocatalytic Antibacterial and Osteoinductive Activities for Implant Infection

Wang and Shi et al.

Supplementary Figures

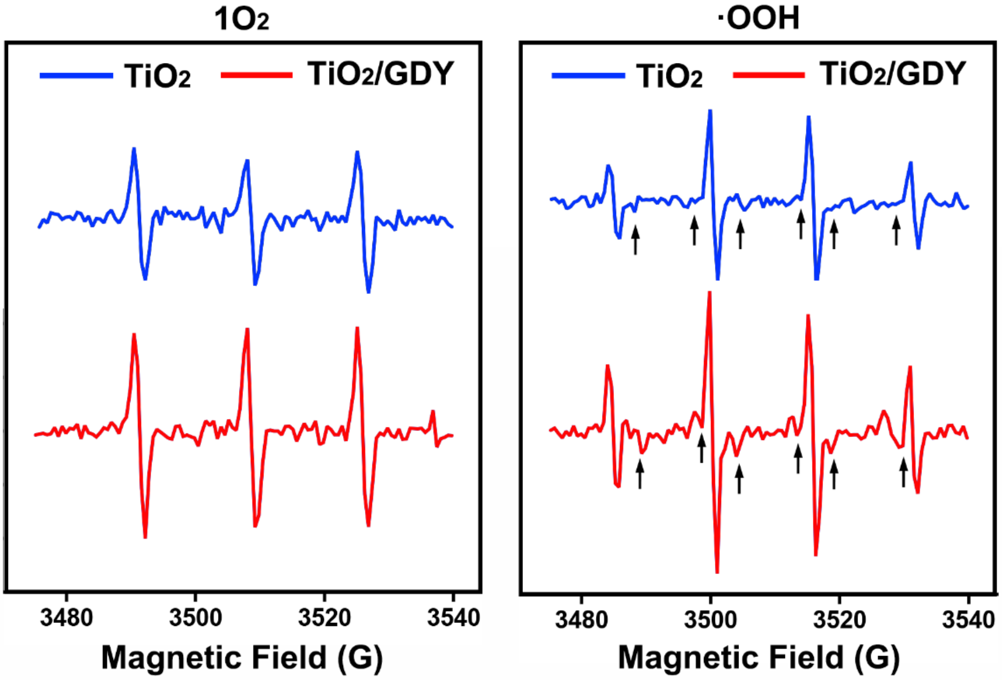

Supplementary Fig. 1 ESR signals for 1O_2 (left) and ·OOH (right) generation in water after TiO_2 (blue) and TiO_2/GDY (red) were irradiated and activated by 300 W Xe lamp. 2,2,6,6-tetramethyl-piperidine (TEMP) and DMPO were used as trapping reagent for 1O2 and ·OOH, respectively. The measurement was performed in water using ESR (Bruke, a300) at room temperature. Black arrows in the right indicate the peaks of ·OOH. Source data are provided as a Source Data file.

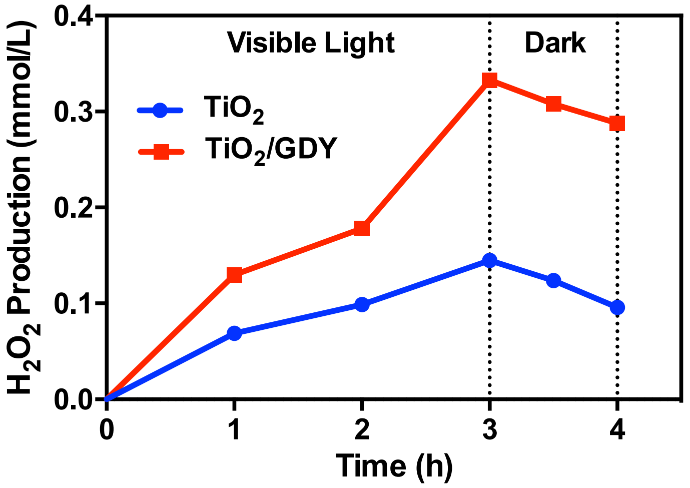

Supplementary Fig. 2 The concentration-time curves of photocatalytic H_2O_2 production in water in the presence of TiO_2 and TiO_2/GDY with 3-hour 300 W Xe lamp irradiation and 1-hour darkness afterwards. The H_2O_2 generation was tested through ferric ion titration method. 15 mg nanofiber photocatalysts in 15 mL H_2O were mixed and stirred in darkness for 30 min, then bubbled with O_2 for 30 min to obtain an O_2 equilibrated environment. After 300 W Xe lamp irradiation, the H_2O_2 concentration in the mixed solution was measured every 1 hour. Also, the H_2O_2 concentration was also measured after turning off the light in dark at 30 and 60 min. Source data are provided as a Source Data file.

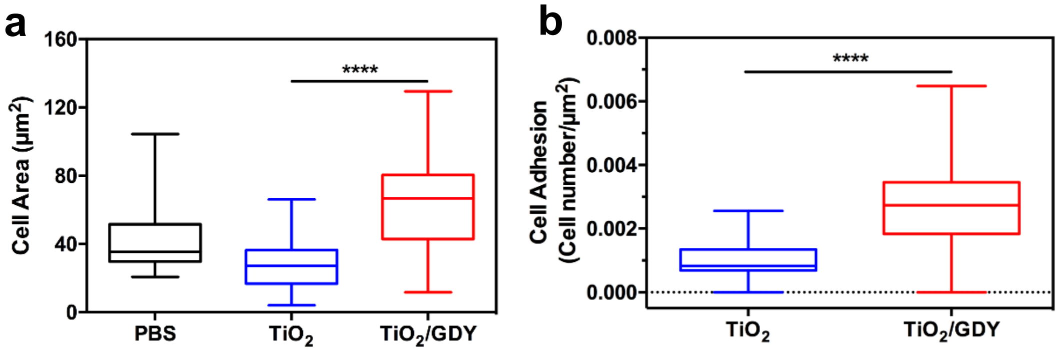

Supplementary Fig. 3 (a) Cell area calculation from Figure. 2a cytoskeleton staining images by Image J (n=25 cells examined over 3 independent experiments). **(b)** Adhered cell number per material area (n =35). The data are shown with medians and quartiles between maximums and minimums (one-way ANOVA was applied for statistical analysis, ****$p<0.0001$). The distribution of cell areas showed that cells on TiO_2/GDY was more spreading than on TiO_2. Cell calculation by DAPI staining exhibited there were significantly more cells adhered on TiO_2/GDY than on TiO_2 per area. Source data are provided as a Source Data file.

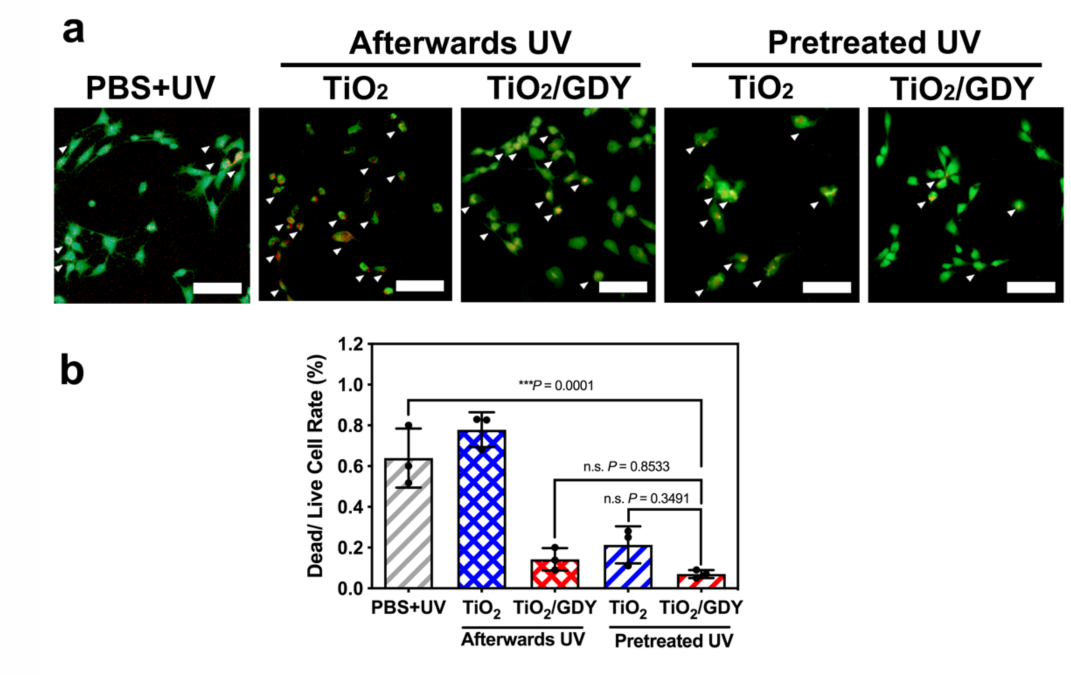

Supplementary Fig. 4 Live/dead staining of cells treated with UV irradiated nanofibers. "Afterwards UV" represents nanofibers added to MC3T3-E1 cells then received UV irradiation together with cells, while "Pretreated UV" represents nanofibers received UV irradiation before added to cells. **(a)** Calcein AM (green) for live cells and PI (red) for dead and apoptosis cells. White arrowheads indicate cells close to death with PI staining in nucleus. (scale bar = 100 μm). **(b)** Semi-quantitative analysis of the live/dead staining. The data are shown as mean ± S. D., error bars = Standard Deviation (n=3 independent experiments) (one-way ANOVA was applied for statistical analysis, $*p<0.05$, $** p<0.01$, $***p<0.001$). Source data of (b) are provided as a Source Data file.

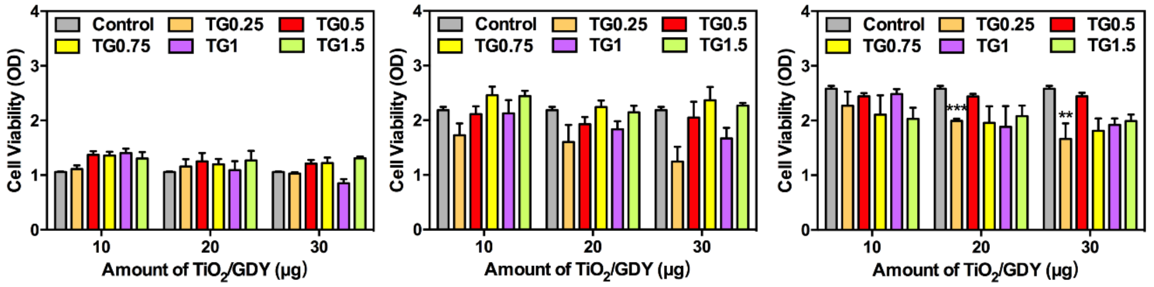

Supplementary Fig. 5 Cell proliferation assay of different ratios of TiO_2/GDY *in vitro*. (a) For CCK-8 analysis, MC3T3-E1 cells cultivated with different ratios of TiO_2/GDY (TG0.25, TG0.5, TG0.75, TG1, TG1.5) and PBS as control for 1, 3, 5 days from left to right. The data are shown as mean ± S. D. Error bars = Standard Deviation (n=3 independent experiments). Source data are provided as a Source Data file.

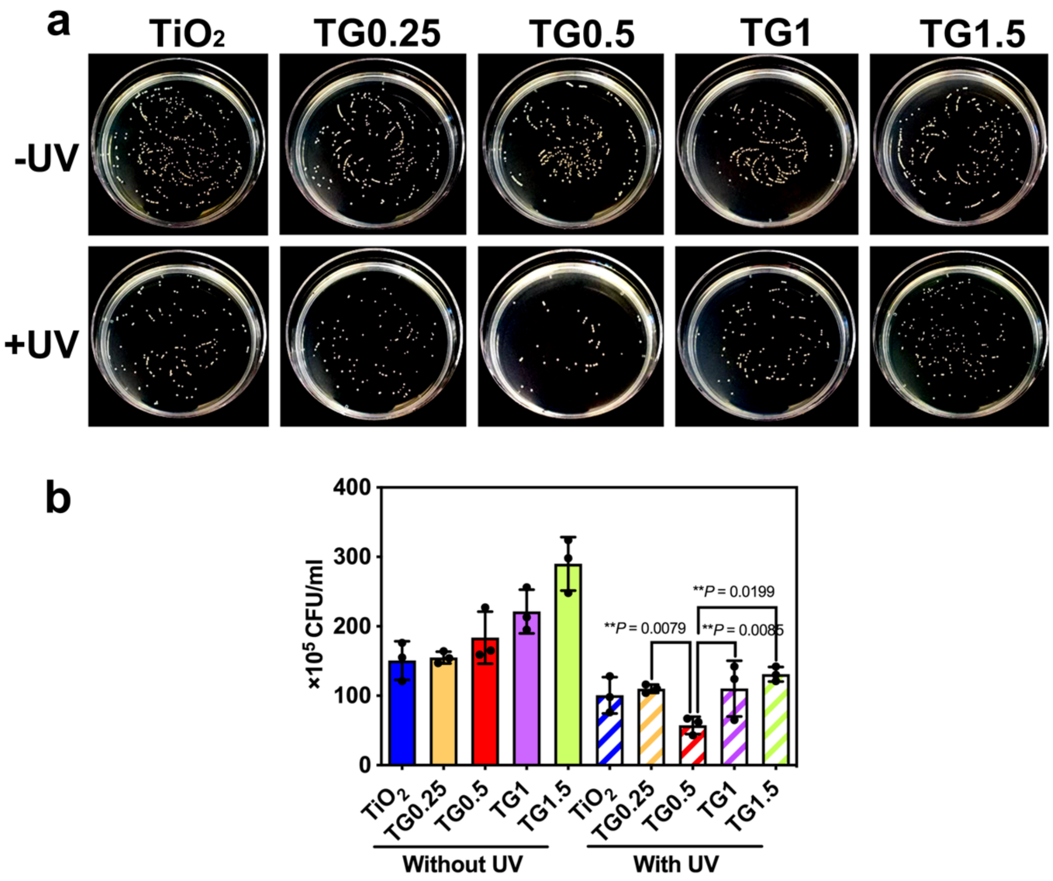

Supplementary Fig. 6 Photocatalytic antibacterial properties of different ratios of TiO_2/GDY *in vitro.* MRSA treated with TiO_2 or different ratios of TiO_2/GDY (TG0.25, TG0.5, TG1, TG1.5) nanofibers, with or without UV (365 nm, 2mW cm^{-1}) irradiation. **(a, b)** Photo and quantitative analysis of bacteria colony of MRSA and the data in b) are shown as mean ± S. D., error bars = Standard Deviation (n =3 independent experiments, one-way ANOVA was applied for statistical analysis, *p<0.05, ** p<0.01, ***p<0.001). Source data of (b) are provided as a Source Data file.

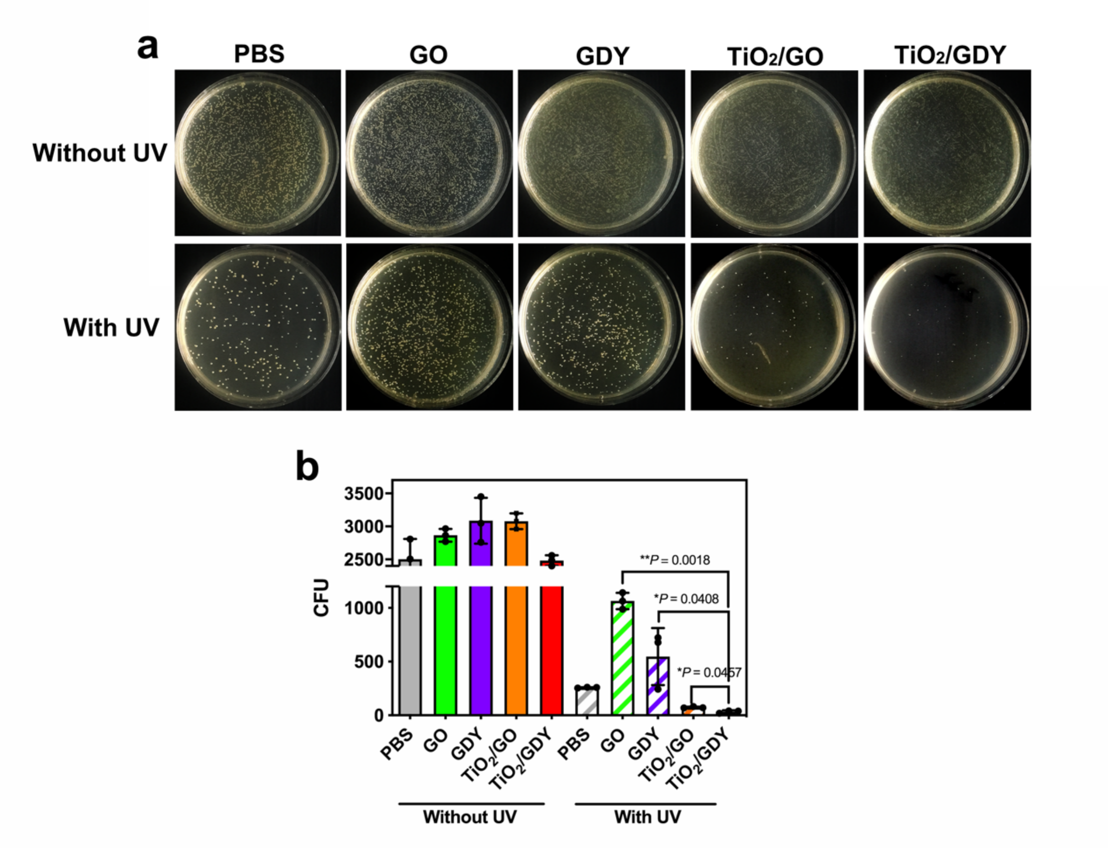

Supplementary Fig. 7 Photocatalytic antibacterial properties of GO, GDY, TiO_2/GO and TiO_2/GDY *in vitro.* MRSA treated with GO, GDY, TiO_2/GO and TiO_2/GDY, with or without UV (365 nm, 2 mW cm^{-1}) irradiation. **(a, b)** Photo and quantitative analysis of bacteria colony of MRSA and the data in b) are shown as mean ± S. D., error bars = Standard Deviation (n =3 independent experiments, one-way ANOVA was applied for statistical analysis, *p<0.05, ** p<0.01, ***p<0.001). Source data of (b) are provided as a Source Data file.

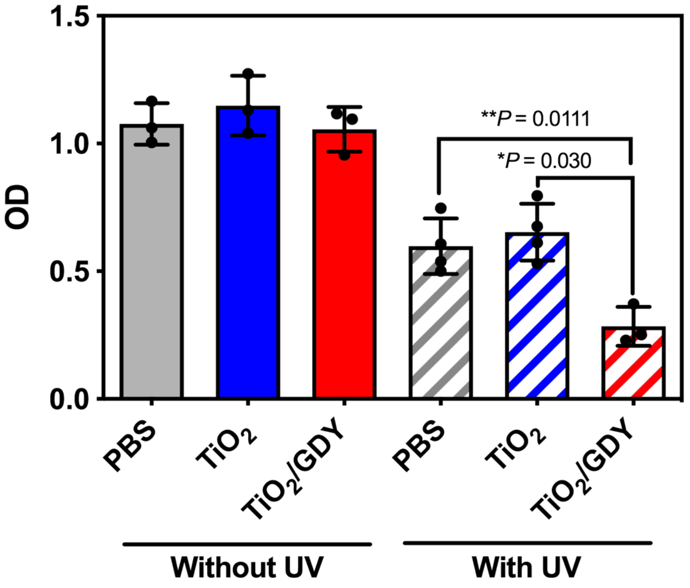

Supplementary Fig. 8 Crystal violet staining of biofilm formation. MRSA treated with TiO_2 or TiO_2/GDY nanofibers then received UV irradiation or not. Absorbance at 590 nm of crystal violet staining of biofilms were measured. Data are shown as mean ± S. D., error bars = Standard Deviation (n =3 independent experiments, one-way ANOVA was applied for statistical analysis, *p<0.05, **p<0.01). Source data are provided as a Source Data file.

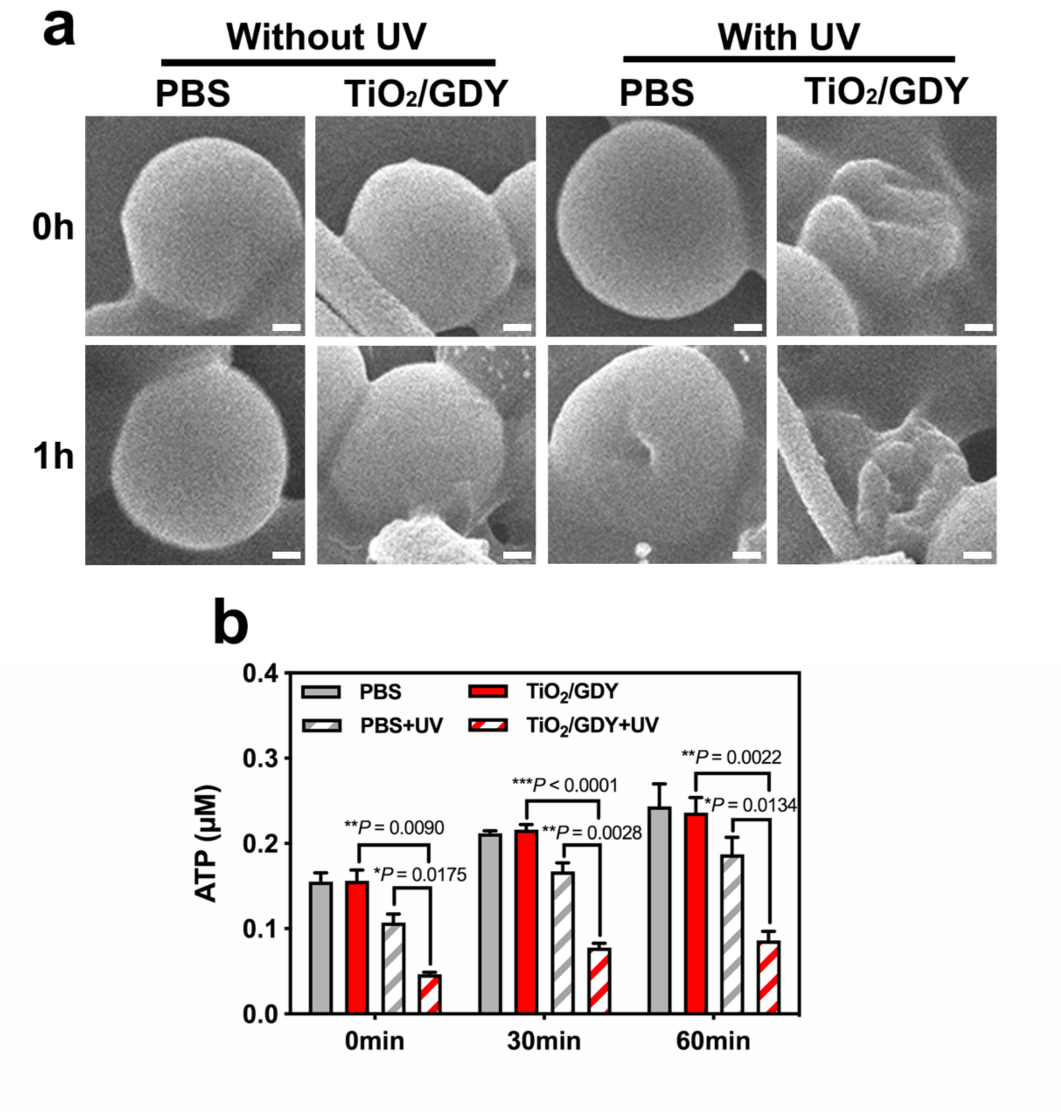

Supplementary Fig. 9 Mechanism in photocatalytic antibacterial effect. (a) SEM images of MRSA after photocatalytic treatment. 10^9 CFU mL^{-1} MRSA treated with TiO_2/GDY nanofibers then received 1 hour of UV irradiation or not. Bacteria were observed immediately (0 h) or incubated in 37 °C for 1 h before observation (1 h). Scale bar = 100 nm. **(b)** ATP level in bacteria after TiO_2/GDY treatment with or without UV irradiation. The ATP levels 0, 30, 60 min after treatment were measured. The data in (b) are shown as mean ± S. D., error bars = Standard Deviation (n =3 independent experiments, two-way ANOVA was applied for statistical analysis, *p<0.05, ** p<0.01, ***p<0.001) Source data of (b) are provided as a Source Data file.

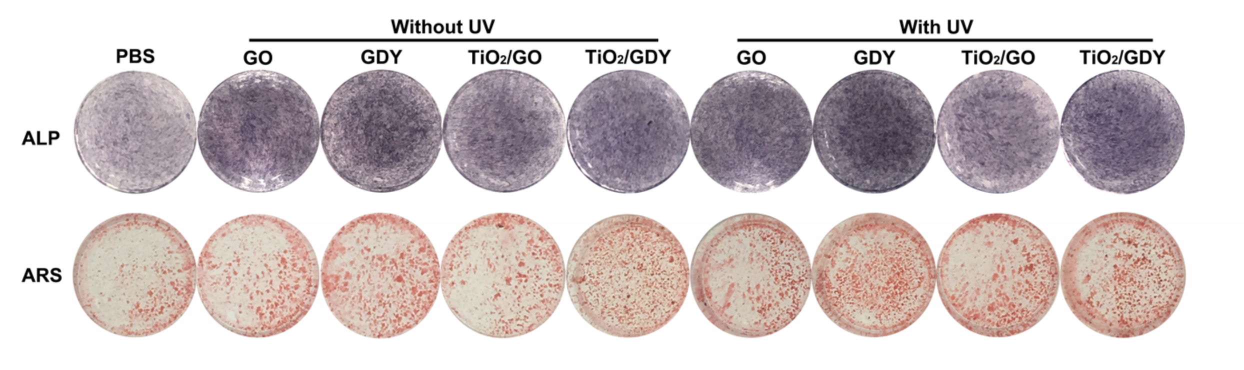

Supplementary Fig. 10 ALP and ARS Staining of MC3T3-E1 cells *in vitro*. Cells were treated with GO, GDY, TiO_2/GO and TiO_2/GDY, with or without UV (365 nm, 2 mW cm^{-1}) irradiation. After 14-day osteogenic induction, ALP staining (upper) and Alizarin red staining (lower) were conducted.

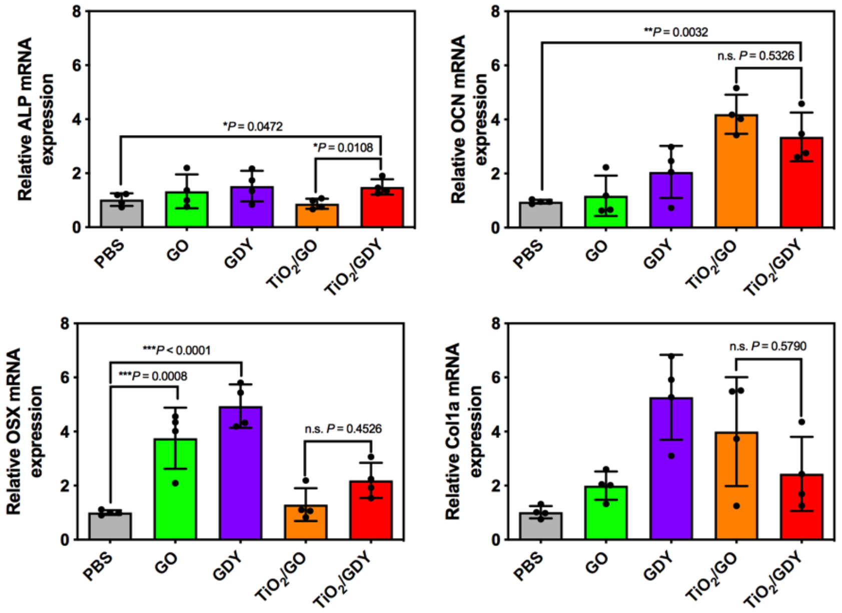

Supplementary Fig. 11 Real time qPCR analysis on expression of Alp, Ocn, Osx and Col1a levels in MC3T3-E1 *in vitro.* MC3T3-E1 cells treated with GO, GDY, TiO_2/GO and TiO_2/GDY with 7-day osteogenic induction. Data are shown as mean ± S. D., error bars = Standard Deviation (n=4 independent experiments, one-way ANOVA was applied for statistical analysis, *p<0.05, ** p<0.01, ***p<0.001, n. s.= not significant). Source data are provided as a Source Data file.

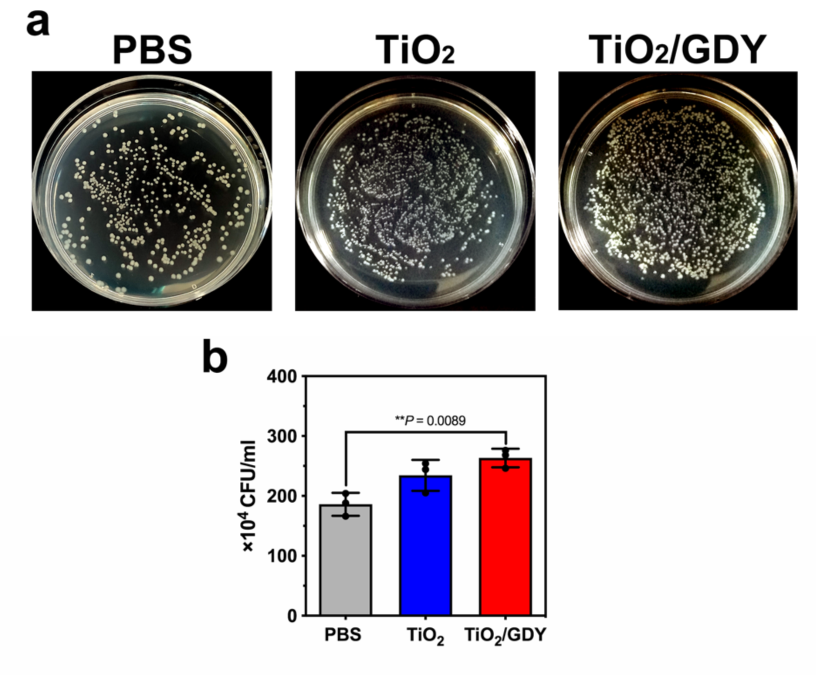

Supplementary Fig. 12 Antibacterial effect of infected TiO_2 and TiO_2/GDY in the mouse implant-associated infection model without UV irradiation. (a, b) Photographs and quantitative analysis of the MRSA bacterial colonies of the infected femurs treated with TiO_2, TiO_2/GDY or PBS as control 5 days after surgery, without UV irradiation. The data are shown as mean ± S. D., error bars = Standard Deviation (n =3 independent experiments, one-way ANOVA was applied for statistical analysis, *p<0.05). Source data of (b) are provided as a Source Data file.

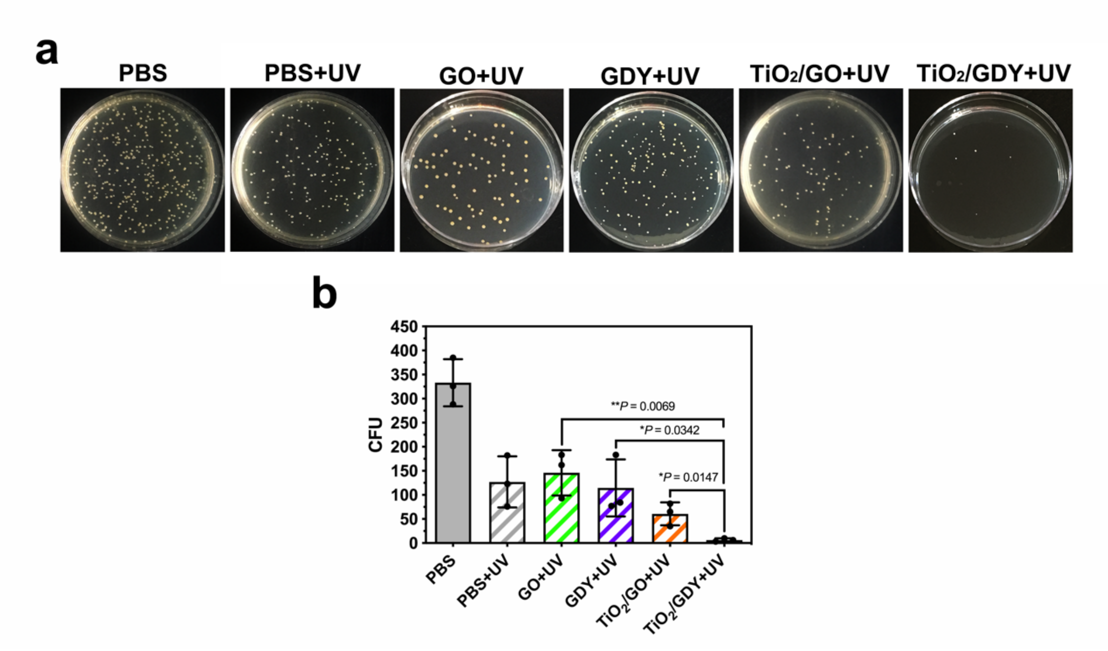

Supplementary Fig. 13 Antibacterial effect of infected GO, GDY, TiO_2/GO and TiO_2/GDY in the mouse implant-associated infection model with UV irradiation. (a, b) Photographs and quantitative analysis of the MRSA bacterial colonies in the defected femurs 5 days after implanted with MRSA infected GO, GDY, TiO_2/GO, TiO_2/GDY or PBS with UV irradiation. The data are shown as mean ± S. D., error bars = Standard Deviation (n =3 biological independent samples, one-way ANOVA was applied for statistical analysis, *$p<0.05$, ** $p<0.01$). Source data of (b) are provided as a Source Data file.

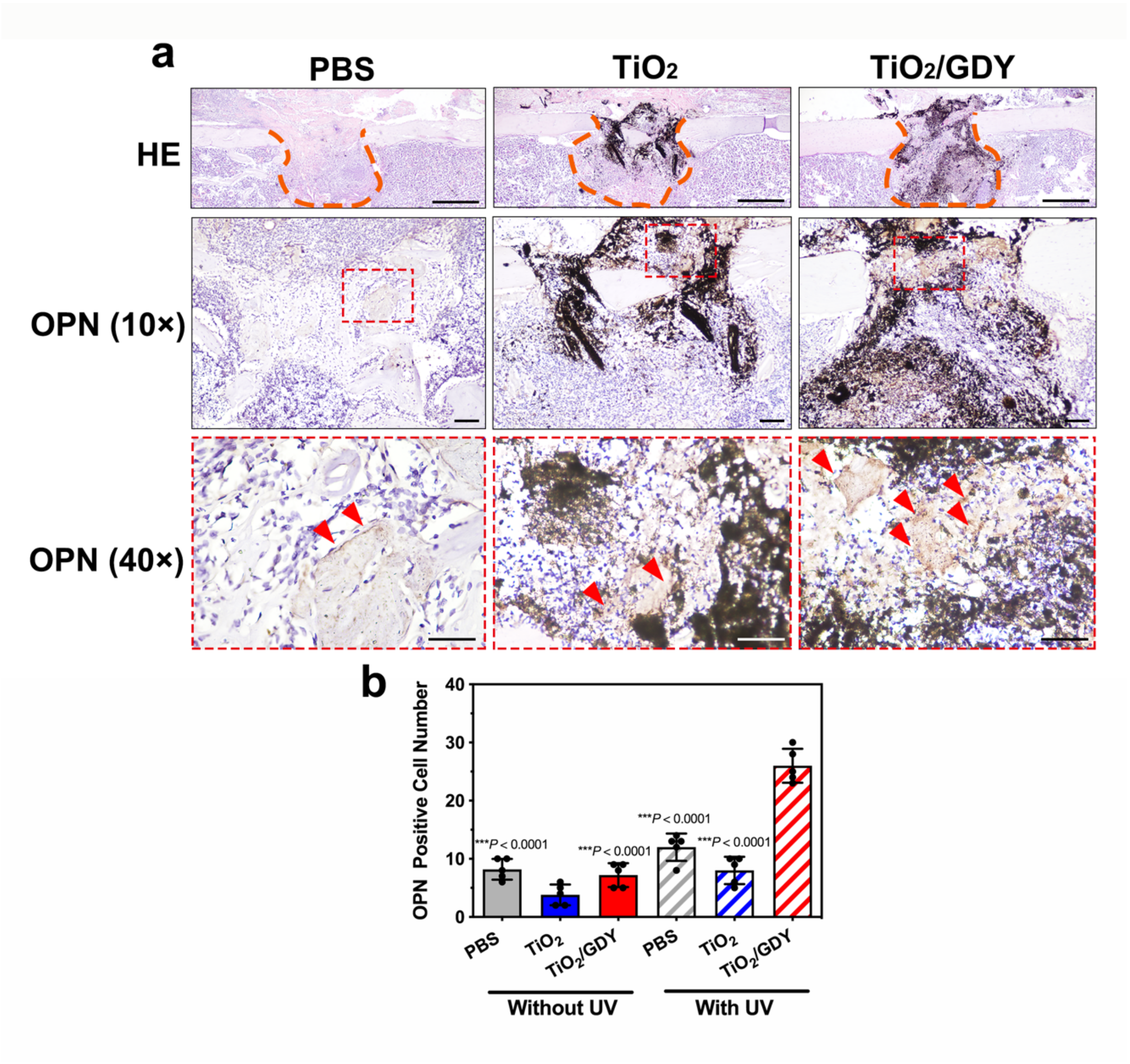

Supplementary Fig. 14 Histomorphological analysis of infected TiO_2 and TiO_2/GDY in the mouse implant-associated infection model without UV irradiation. (a) H&E staining and immunohistochemical analysis of OPN images for infection and bone formation after implanted with MRSA infected TiO_2 or TiO_2/GDY nanofibers 5 days, without UV irradiation. Orange dot lines in H&E images represent infectious and necrotic areas (scale bar = 500 μm). OPN 10×: scale bar = 100 μm ; OPN 40×: scale bar = 50 μm; red arrowheads point out OPN positive osteoblasts. **(b)** Counting of OPN positive cell numbers in the IHC images of both UV and without UV groups. The data are shown as mean ± S. D., error bars = Standard Deviation (n=5 biological independent samples) (all groups were compared to TiO_2/GDY+UV group for statistical analysis, one-way ANOVA was applied for statistical analysis, *p<0.05, ** p<0.01, ***p<0.001). Source data of (b) are provided as a Source Data file.

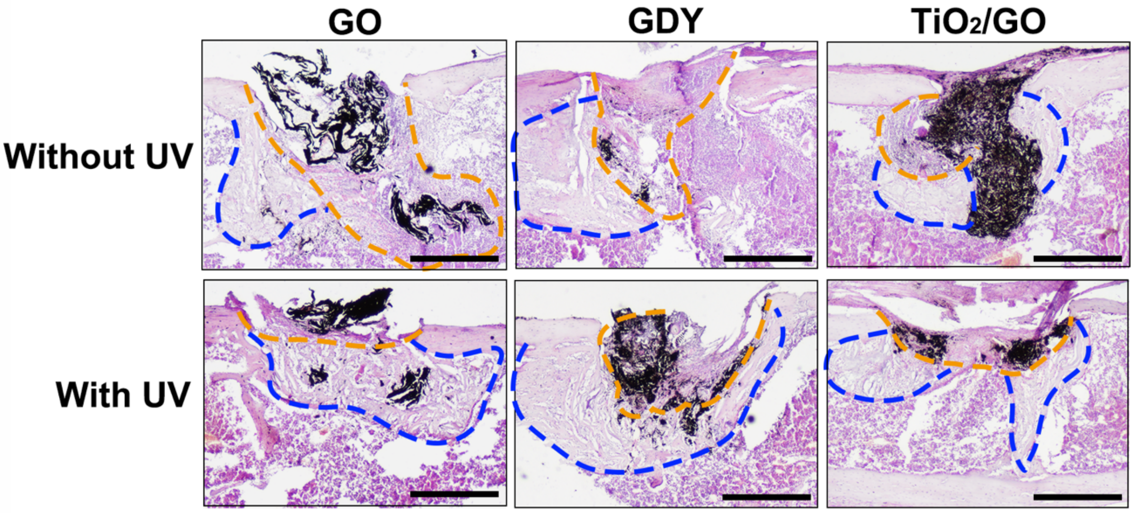

Supplementary Fig. 15 H&E staining of infected GO, GDY and TiO_2/GO in the mouse implant-associated infection model with or without UV irradiation. Images for infection and bone formation 5 days after implanted with MRSA infected GO, GDY or TiO_2/GO with or without UV irradiation. Orange dotted lines represent infectious and necrotic areas; between the orange and blue dotted lines are new bone formation areas (n =3) (scale bar = 500 μm).

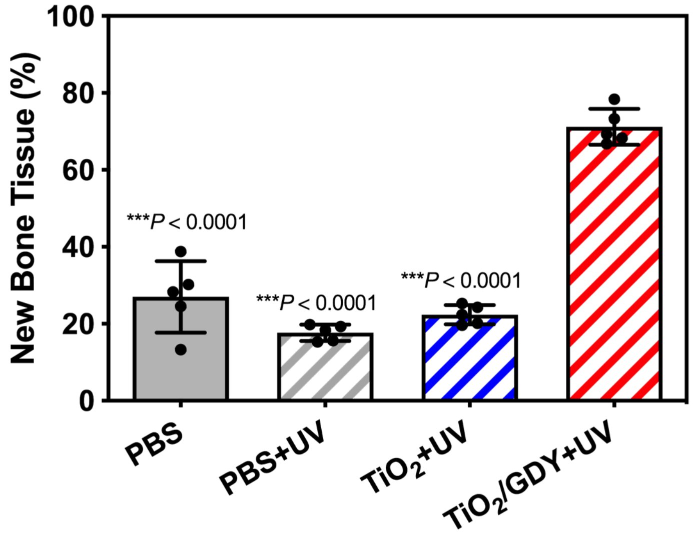

Supplementary Fig. 16 Histomorphological analysis of bone regeneration 4 weeks after implement of mouse implant-associated infection model. (a) Semi-quantitative assay of new bone formation with the percentage of blue area in the dot-lined boxed on Masson staining images. The data are shown as mean ± S. D., error bars = Standard Deviation (n =5 biological independent samples, one-way ANOVA was applied for statistical analysis, * denotes the significant difference compared to TiO_2/GDY group p<0.05, ** p<0.01, ***p<0.001). Source data are provided as a Source Data file.

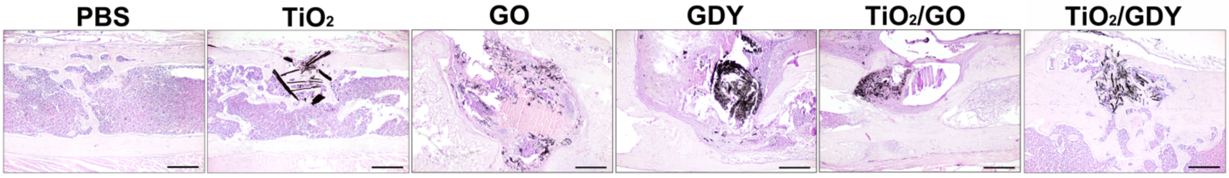

Supplementary Fig. 17 H&E staining of bone regeneration 4 weeks after implement of mouse implant-associated infection model. Femurs were implanted with MRSA infected GO, GDY, TiO_2/GO， TiO_2, TiO_2/GDY, or PBS as control with prior UV irradiation. Scale bar = 500 μm. (n=3 biological independent samples)

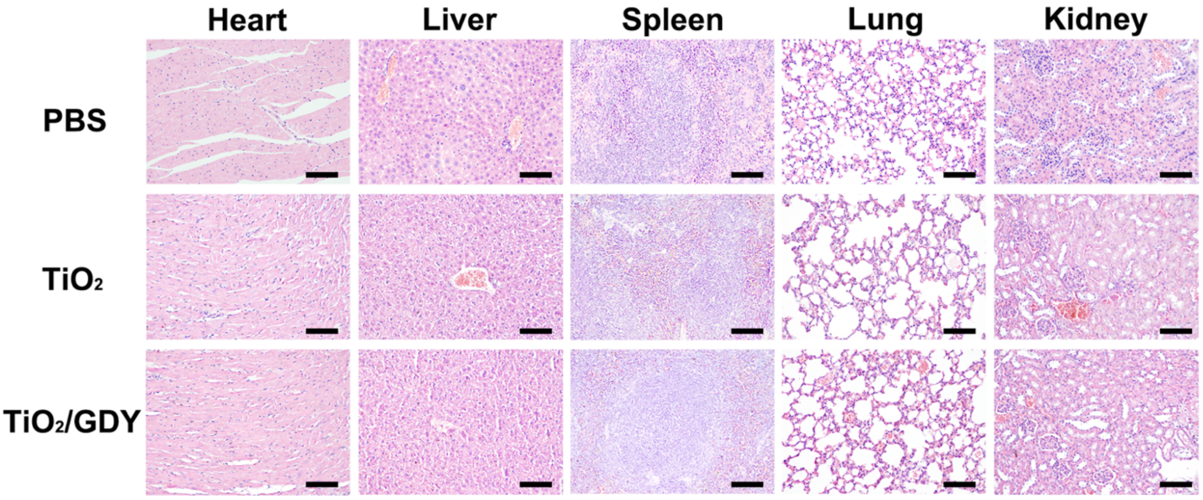

Supplementary Figure. 18 H&E staining of organs in mouse implant-associated infection model. Heart, liver, spleen, lung and kidney in mice implanted with TiO_2 or TiO_2/GDY nanofibers for 4 weeks. PBS as the control group. (n = 3) (scale bar = 100 μm).
