# Supplementary material for: Graphdiyne-modified TiO2 nanofibers with osteoinductive and enhanced photocatalytic antibacterial activities to prevent implant infection
Source: Nat Commun. 2020 Sep 8;11:4465. doi: 10.1038/s41467-020-18267-1 (PMC7479592; doi:10.1038/s41467-020-18267-1)
Supplement: Supplementary file 2 — Reporting Summary [file 41467_2020_18267_MOESM2_ESM.pdf]

nature research

Corresponding author(s): Jiaguo Yu, Yufeng Zhang

Last updated by author(s): Jul 29, 2020

Reporting Summary

Nature Research wishes to improve the reproducibility of the work that we publish. This form provides structure for consistency and transparency in reporting. For further information on Nature Research policies, see Authors & Referees and the Editorial Policy Checklist .

Statistics

For all statistical analyses, confirm that the following items are present in the figure legend, table legend, main text, or Methods section.

n/a	Confirmed	
☐	☒	The exact sample size (*n*) for each experimental group/condition, given as a discrete number and unit of measurement
☐	☒	A statement on whether measurements were taken from distinct samples or whether the same sample was measured repeatedly
☐	☒	The statistical test(s) used AND whether they are one- or two-sided<br>*Only common tests should be described solely by name; describe more complex techniques in the Methods section.*
☒	☐	A description of all covariates tested
☒	☐	A description of any assumptions or corrections, such as tests of normality and adjustment for multiple comparisons
☐	☒	A full description of the statistical parameters including central tendency (e.g. means) or other basic estimates (e.g. regression coefficient) AND variation (e.g. standard deviation) or associated estimates of uncertainty (e.g. confidence intervals)
☐	☒	For null hypothesis testing, the test statistic (e.g. *F*, *t*, *r*) with confidence intervals, effect sizes, degrees of freedom and *P* value noted<br>*Give P values as exact values whenever suitable.*
☒	☐	For Bayesian analysis, information on the choice of priors and Markov chain Monte Carlo settings
☒	☐	For hierarchical and complex designs, identification of the appropriate level for tests and full reporting of outcomes
☒	☐	Estimates of effect sizes (e.g. Cohen's *d*, Pearson's *r*), indicating how they were calculated

Our web collection on statistics for biologists contains articles on many of the points above.

Software and code

Policy information about availability of computer code

Data collection	Titan G2 60-300 for TEM images, Renishaw Co. RM1000 for Raman spectroscopy, Powereach® JC2000C1 for contact angel measurement, SOPTOP, UV2800 for ultraviolet spectrophotometer, Bruke emx nano and Bruke a300 for ESR, Leica-LCS-SP8-STED for laser scanning confocal micrograph, TESCAN VEGA 3 LMU for SEM, Zeiss Axio Imager A2 for fluorescence micrograph, PowerWave XS2 BioTek and Molecular Devicesmd, spectramax i3x for absorbance measurement
Data analysis	Graphpad Prism for statistical analysis, Image J for cell counting and cell area measurement

For manuscripts utilizing custom algorithms or software that are central to the research but not yet described in published literature, software must be made available to editors/reviewers. We strongly encourage code deposition in a community repository (e.g. GitHub). See the Nature Research guidelines for submitting code & software for further information.

Data

Policy information about availability of data

All manuscripts must include a data availability statement. This statement should provide the following information, where applicable:

- Accession codes, unique identifiers, or web links for publicly available datasets
- A list of figures that have associated raw data
- A description of any restrictions on data availability

The experiment data that support the findings of this study are available from the corresponding author upon reasonable request. The source data underlying Figs. 1b-f, Fig. 2e, f, Fig. 3b, d, f, Fig. 4b-d, Fig. 5b and Supplementary Figs. 1, 2, 3, 4b, 5, 6b, 7b, 8, 9b, 11, 12b, 13b, 14b, 16 are provided as a Source Data file in the final part.

Field-specific reporting

Please select the one below that is the best fit for your research. If you are not sure, read the appropriate sections before making your selection.

- [x] Life sciences
- [ ] Behavioural & social sciences
- [ ] Ecological, evolutionary & environmental sciences

For a reference copy of the document with all sections, see nature.com/documents/nr-reporting-summary-flat.pdf

Life sciences study design

All studies must disclose on these points even when the disclosure is negative.

Sample size	No sample size calculation was performed. Sample size was based on capable of statistical analysis and described in figure legend.
Data exclusions	No data were excluded.
Replication	Each experiment was performed 3-5 independent replications and was described in figure legend.
Randomization	Animals were randomly grouped for operation. Cell samples and bacteria were randomly grouped for experiments. Enough replications and control groups were carried out in consistent environment to control covariates.
Blinding	Blinding was not applied but three investigators were involved in animal surgeries. Because materials and operation steps were different in each group.

Reporting for specific materials, systems and methods

We require information from authors about some types of materials, experimental systems and methods used in many studies. Here, indicate whether each material, system or method listed is relevant to your study. If you are not sure if a list item applies to your research, read the appropriate section before selecting a response.

Materials & experimental systems

n/a	Involved in the study	
☐	☒	Antibodies
☐	☒	Eukaryotic cell lines
☒	☐	Palaeontology
☐	☒	Animals and other organisms
☒	☐	Human research participants
☒	☐	Clinical data

Methods

n/a	Involved in the study	
☒	☐	ChIP-seq
☒	☐	Flow cytometry
☒	☐	MRI-based neuroimaging

Antibodies

Antibodies used	OPN monoclonal antibody, Santa cruz sc-21742
Validation	The antibody was validated by manufacturer through Western blot and Immunoperoxidase staining.

Eukaryotic cell lines

Policy information about cell lines

Cell line source(s)	The cell line MC3T3-E1 used in this study was from ThermoScientific, United States.
Authentication	None of the cell lines used were authenticated.
Mycoplasma contamination	The cell line used in the study was negative for mycoplasma contamination.
Commonly misidentified lines (See ICLAC register)	The cell line was not misidentified.

Animals and other organisms

Policy information about studies involving animals; ARRIVE guidelines recommended for reporting animal research

Laboratory animals	Kunming mice, 8-week old, female. Ambient temperature 20-25°C, humidity 40-50% and daylight 12h/day.

Wild animals	The study did not involve wild animals
Field-collected samples	The study did not involve samples collected from the field.
Ethics oversight	All animal surgical procedures were approved by the Ethics Committee for Animal Research of Wuhan University in China.

Note that full information on the approval of the study protocol must also be provided in the manuscript.
